# DHX36 Enhances RIG-I Signaling by Facilitating PKR-Mediated Antiviral Stress Granule Formation

**DOI:** 10.1371/journal.ppat.1004012

**Published:** 2014-03-20

**Authors:** Ji-Seung Yoo, Kiyohiro Takahasi, Chen Seng Ng, Ryota Ouda, Koji Onomoto, Mitsutoshi Yoneyama, Janice Ching Lai, Simon Lattmann, Yoshikuni Nagamine, Tadashi Matsui, Kuniyoshi Iwabuchi, Hiroki Kato, Takashi Fujita

**Affiliations:** 1 Laboratory of Molecular Genetics, Institute for Virus Research, Kyoto University, Kyoto, Japan; 2 Laboratory of Molecular Cell Biology, Graduate School of Biostudies, Kyoto University, Kyoto, Japan; 3 Institute for Innovative NanoBio Drug Discovery and Development, Graduate School of Pharmaceutical Science, Kyoto University, Kyoto, Japan; 4 Division of Molecular Immunology, Medical Mycology Research Center, Chiba University, Chuo-ku, Chiba, Japan; 5 Friedrich Miescher Institute for Biomedical Research, Basel, Switzerland; 6 Department of Biochemistry I, School of Medicine, Kanazawa Medical University, Uchinada, Ishikawa, Japan; Harvard Medical School, United States of America

## Abstract

RIG-I is a DExD/H-box RNA helicase and functions as a critical cytoplasmic sensor for RNA viruses to initiate antiviral interferon (IFN) responses. Here we demonstrate that another DExD/H-box RNA helicase DHX36 is a key molecule for RIG-I signaling by regulating double-stranded RNA (dsRNA)-dependent protein kinase (PKR) activation, which has been shown to be essential for the formation of antiviral stress granule (avSG). We found that DHX36 and PKR form a complex in a dsRNA-dependent manner. By forming this complex, DHX36 facilitates dsRNA binding and phosphorylation of PKR through its ATPase/helicase activity. Using DHX36 KO-inducible MEF cells, we demonstrated that DHX36 deficient cells showed defect in IFN production and higher susceptibility in RNA virus infection, indicating the physiological importance of this complex in host defense. In summary, we identify a novel function of DHX36 as a critical regulator of PKR-dependent avSG to facilitate viral RNA recognition by RIG-I-like receptor (RLR).

## Author Summary

Cellular responses to environmental stress are critical for maintaining homeostasis in living organisms. In one type of response, eukaryotic cells exhibit rapid formation of aggregates with RNA and multiple RNA-binding proteins in the cytoplasm termed stress granules (SGs). Over the past decade, SGs have been suggested to be important compartments and play essential roles in cellular stress responses. We have previously reported that virus infection induced SG-like aggregates and are crucial for antiviral response, therefore termed them as antiviral (av) SGs. In this report, we describe a novel function of DExD/H box RNA helicase 36 (DHX36), as a critical activator of double-stranded RNA (dsRNA)-dependent protein kinase (PKR), which directly trigger avSG formation in virus-infected cells. Our results reveal a novel link between DHX36 and avSG which functions as a platform to facilitate sensing of viral invasion and triggering antiviral responses.

## Introduction

Cellular responses against various stresses are crucial for maintaining homeostasis. Virus infection is one class of cellular stress that induces a number of host responses through the activation of pattern recognition receptors (PRRs), which detect pathogen-associated molecular patterns (PAMPs) with high specificity for protection against microbial invasion. RIG-I, a DExD/H-box RNA helicase family member, functions as a cytosolic viral RNA sensor, recognizes specific structures of non-self RNAs, including double-stranded RNA (dsRNA) and *in vitro* transcripts containing 5′ppp- and partial double-stranded structure due to copy back (5′ppp-cbRNA) [1], [2]. Upon sensing non-self RNA, RIG-I promptly induces type I interferon (IFN) and other cytokines to provide a primary defense program against viruses [3].

In addition to RIG-I, interferon-inducible dsRNA-dependent protein kinase (PKR) represents another cytosolic foreign RNA sensor and plays a pivotal role in regulating innate immune responses [4], [5], [6], [7], [8], [9]. Both RIG-I and PKR exert an antiviral effect by detecting particular RNA species such as dsRNA or secondary-structured RNA, which can be generated during replication of both RNA and DNA viruses [2], [10]. Although accumulating evidence has suggested that PKR has an important role in antiviral host defense, the exact mechanisms underlying the regulation of innate immunity remain to be elucidated.

Recently, we and others have shown that PKR is a key factor for the induction of cytoplasmic bodies called antiviral stress granules (avSGs) by viral infection, and we further clarified that SGs provide a critical platform for interactions between antiviral proteins and non-self RNA ligands [11], [12], [13], [14]. Increasing evidence has clearly shown a tight link between SGs and antiviral innate immunity and, in fact, most viruses appear to antagonize SG formation through multiple strategies for the evasion of host antiviral responses [15], [16].

Previous studies have suggested the involvement of various DExD/H-box RNA helicases in innate immunity by either direct sensing of various PAMPs or functioning as an adaptor molecule in the signaling pathway [17]. DExD/H-box helicase 36 (DHX36), also termed RNA helicase associated with AU-rich RNA element (ARE) (RHAU) is implicated as a factor in ARE-mediated mRNA decay [18]. DHX36 can directly bind and unwind the specific structure of guanine-tetramolecular quadruplex-DNA or -RNA (G4-DNA or G4-RNA) through its amino-terminal RHAU-specific motif together with helicase activity [19], [20]. Recently, DHX36 has also been characterized as a critical factor for host immune responses. For instance, DHX36 was shown to function as a foreign DNA sensor in plasmacytoid dendritic cells (pDC) [21]. More recently DHX36 has been suggested to play a pivotal role in the IFN signaling pathway by forming a complex together with other RNA helicases for dsRNA sensing [22]. Furthermore, it has also been demonstrated that DHX36 localizes in stress granules under various cellular stress conditions [23]. These results prompted us to investigate whether DHX36 has the potential to regulate innate immunity by interacting with antiviral proteins in virus-induced stress granules.

In this report, we examined the role of DHX36 in the host innate immune response by virus infection and non-self RNA ligand transfection in fibroblast cells. It was found that DHX36 regulates innate immunity by facilitating virus-induced stress granule formation and contributes to PKR activation by viral RNA.

## Results

### DHX36 Regulates Innate Immunity in a Stimulus-Dependent Manner

**Figure 1:**
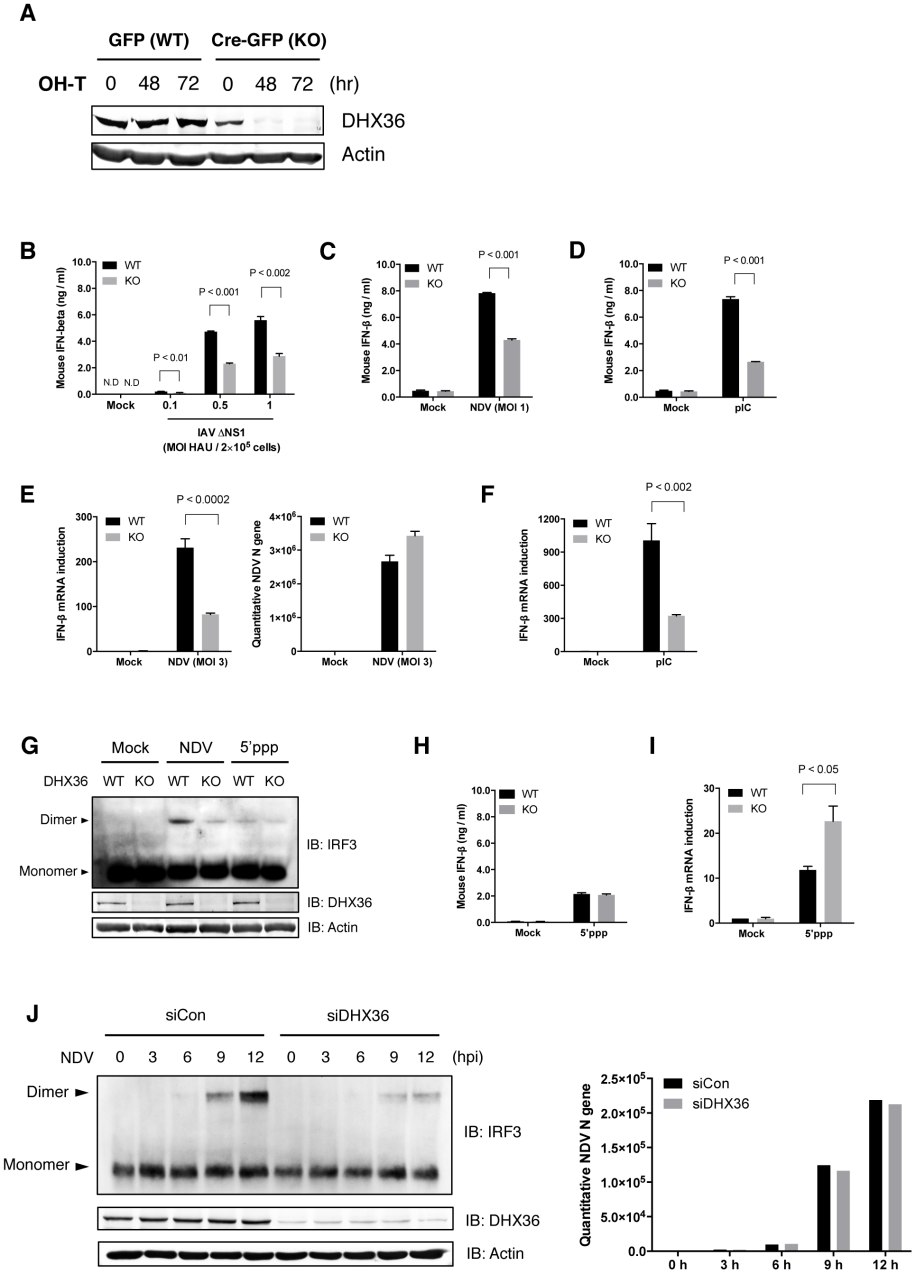
Critical role of DHX36 in virus-induced activation of IFN-ß gene. (**A**) MEF cells derived from mice with knock-in lox/*dhx36*/lox gene either with transgene for tamoxifen-inducible GFP (WT) or Cre-GFP (KO) were treated with 1 µM tamoxifen. After incubation for the indicated times, DHX36 protein level was examined by immunoblotting. (**B–D**) After 72 h treatment with tamoxifen, cells were infected with IAVΔNS1 (**B**), NDV (**C**) or transfected with poly I∶C (pIC) (**D**) as indicated. After 16 h infection or transfection, culture media were subjected to ELISA to determine IFN-ß protein level. (**E–F**) DHX36 WT or KO-induced MEF cells were infected with NDV (**E**), or transfected with pIC (**F**). After 9 h incubation, cells were harvested and total RNA was collected. cDNA was synthesized with random hexamers as a primer. Level of NDV replication and IFN-ß gene induction was evaluated by real-time qPCR. (**G**) Cell extracts from mock-treated, NDV-infected, or 5′ppp-transfected DHX36 WT or KO cells were subjected to native-PAGE and IRF-3 dimer was detected by immunoblotting using anti-mouse IRF-3 antibody. (**H–I**) DHX36 WT or KO-induced MEF cells were mock-treated or transfected with 5′ppp-cbRNA (5′ppp). After 16 h transfection, culture media were subjected to ELISA to determine IFN-ß protein level (**H**). After 9 h incubation, cells were harvested and total RNA was collected to evaluate IFN-ß gene induction by real-time qPCR **(I)**. (**J**) Control and DHX36 knocked-down HEK293T cells were mock-treated or infected with NDV. Whole-cell lysates at the indicated times were subjected to either Native-PAGE to examine the level of IRF-3 dimerization or SDS-PAGE to evaluate the protein level of DHX36 and actin by Western blot analysis (left). Level of NDV replication was confirmed by real-time qPCR (right). Viruses: IAVΔNS1, Influenza virus with NS1 gene deletion; NDV, Newcastle disease virus. Data are the mean ± standard error of the mean (SEM), P value by Student's t test is indicated.

To further evaluate the function of DHX36 in the RLR-mediated pathway, we employed the conditional *dhx36* KO system [24]. First, the efficiency of tamoxifen-induced deletion of the *dhx36* gene in MEFs was examined (Figure 1A). After 72 hours of tamoxifen treatment, DHX36 protein became undetectable. Next, the effect of *dhx36* KO on IFN-ß gene activation was examined. IFN-ß induction by infection with influenza A virus (IAV) ΔNS1, Newcastle disease virus (NDV) or by transfection of poly rI: poly rC (pIC) was significantly reduced in the absence of DHX36 as revealed by quantification of IFN-ß protein (Figure 1B–D) and mRNA (Figure 1E and Figure 1F). Moreover, DHX36 is required for efficient IRF-3 dimerization in NDV-infected cells (Figure 1G). In summary, these results reveal the involvement of DHX36 in virus-induced signaling for IFN-ß gene activation.

It was reported that 5′ppp-cbRNA activates RIG-I [25], [26]. Interestingly, IFN-ß production, as well as IRF3 dimerization, induced by transfection of 5′ppp-cbRNA did not change in the presence or absence of DHX36 (Figure 1G and Figure 1H). Whereas IFN-ß mRNA level was increased in the absence of DHX36 (Figure 1I). Because these IFN-ß mRNAs are likely polyadenylated (Figure S1), a possible involvement of DHX36 in posttranscriptional regulation is suggested [18]. However we focus on the function of DHX36 on the virus-induced signaling in this report.

In NDV-infected HEK293T cells, knockdown of DHX36 profoundly diminished active IRF-3 dimers (Figure 1J). Since NDV replication was comparable up to 12 hr (Figure 1E and Figure 1J), in the presence or absence of DHX36, the data suggest the involvement of DHX36 in antiviral signaling through IRF-3. Altogether, these results indicate that DHX36 is involved in IFN-ß production in a stimulus-dependent manner.

### Co-localization of DHX36, RIG-I and PKR in avSG

**Figure 2:**
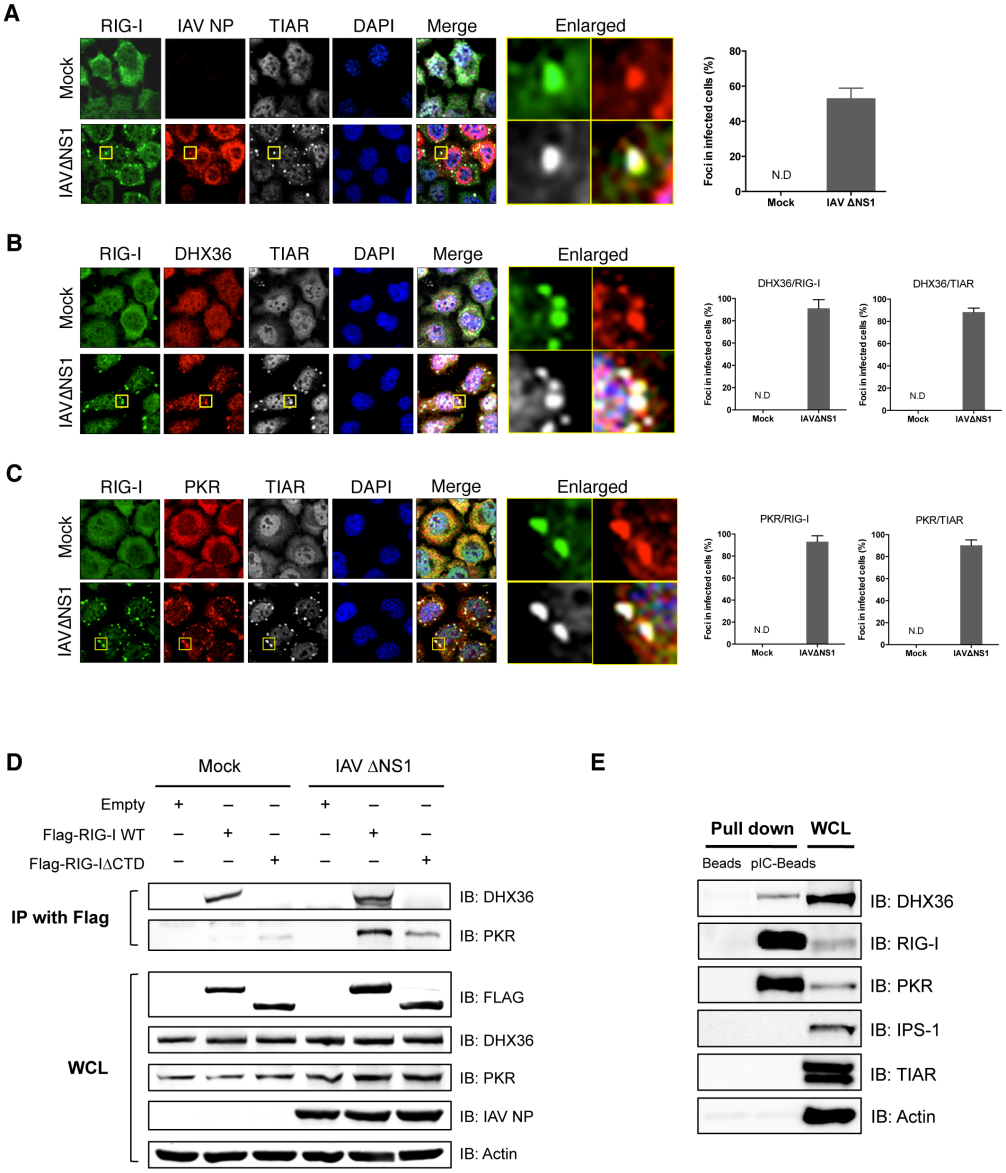
Co-localization and physical interaction of DHX36 with PKR and RIG-I. (**A–C**) HeLa cells were mock-treated or infected with IAVΔNS1. After 12 h infection, cells were fixed and stained for the indicated proteins (Experimental Procedures). Nuclei were visualized by DAPI staining. The images were taken by confocal microscopy. High magnification images of the square area are shown (Enlarged). Cells with foci containing RIG-I, IAV NP, and TIAR were counted by examining of 100 cells (**A**). Cells with foci containing DHX36 and TIAR or DHX36 and RIG-I were counted (**B**). Foci with PKR and TIAR or PKR and RIG-I were counted (**C**). Data are the mean standard ± error of the mean (SEM). (N.D = not detected). (**D**) HEK293T cells transfected with empty vector, expression vector for Flag-tagged RIG-I WT, or Flag-tagged RIG-IΔCTD were mock treated or infected with IAVΔNS1 for 12 h. Whole-cell extracts were prepared and immunoprecipitated with anti-Flag antibody. The precipitates (IP with Flag) were analyzed for DHX36 and PKR by immunoblotting. Protein expression in the whole-cell lysate (WCL) was confirmed by immunoblotting. (**E**) Whole-cell extract from uninfected HeLa cells was precipitated with either empty (Beads) or poly I∶C-conjugated beads (pIC-Beads) in the presence of RNase inhibitor. The precipitates were further subjected to immunoblotting to evaluate the protein-protein interaction using the indicated antibodies.

We recently reported that antiviral stress granules (avSGs) are crucial for both sensing virus infection and type I IFN signaling by providing a critical platform for interaction between antiviral proteins and non-self RNA ligands [11], [13]. DHX36 was reported to localize in SGs induced by various stresses [23]. We used HeLa cells for DHX36 localization by immunostaining because this cell line was used in our previous studies. We confirmed that DHX36 upregulates virus- or pIC-induced signaling in HeLa cells (Figure S2). Infection of HeLa cells with IAVΔNS1 induced speckles of SG marker T-cell intracellular antigen-1-related protein (TIAR) and these speckles also co-localized with RIG-I and IAV nucleocapsid protein (NP) (Figure 2A). Quantification also confirmed that viral infection specifically induced co-localization of these proteins (bar graph in Figure 2A). DHX36 is normally distributed diffusely in both the nucleus and cytoplasm; however, IAVΔNS1 infection induced its re-localization to speckles, which co-localized with RIG-I and TIAR (Figure 2B). Co-localization of DHX36/TIAR and DHX36/RIG-I was highly inducible and was observed in the majority of speckles (∼90%, bar graph in Figure 2B). We also examined co-localization of PKR with RIG-I in avSGs induced by infection with IAVΔNS1 (Figure 2C). These results confirmed that DHX36, RIG-I, PKR and TIAR localizes in avSGs.

### Physical Interaction of DHX36 with RIG-I and PKR

Because DHX36 co-localized with RIG-I and PKR, we examined their physical interaction by co-immunoprecipitation. Cells were transfected with expression vector for full-length RIG-I or RIG-IΔCTD, which lacks the C-terminal domain (CTD). The cells were mock treated or infected with IAVΔNS1 for 12 h and physical interaction was monitored by co-immunoprecipitation. DHX36 interacted with RIG-I regardless of virus infection in its CTD-dependent manner (Figure 2D, Figure S3). However, interaction between PKR and RIG was dependent on virus infection. Moreover, RIG-I CTD was partly responsible for this interaction. To further confirm the interaction of both DHX36 and antiviral proteins with dsRNA, we performed pIC pull-down analysis using an extract from uninfected cells (Figure 2E). The result clearly showed that DHX36 has binding affinity to dsRNA in addition to AU-rich RNA, as reported previously [18]. Purified recombinant DHX36 exhibited clear binding with pIC (Figure S4), demonstrating direct interaction between DHX36 and dsRNA. RIG-I and PKR also bound to the dsRNA, presumably as a complex (Figure 2E). However, TIAR, a SG component and a known RNA binding protein, did not bind to the dsRNA.

### DHX36 Recognizes Viral RNAs

**Figure 3:**
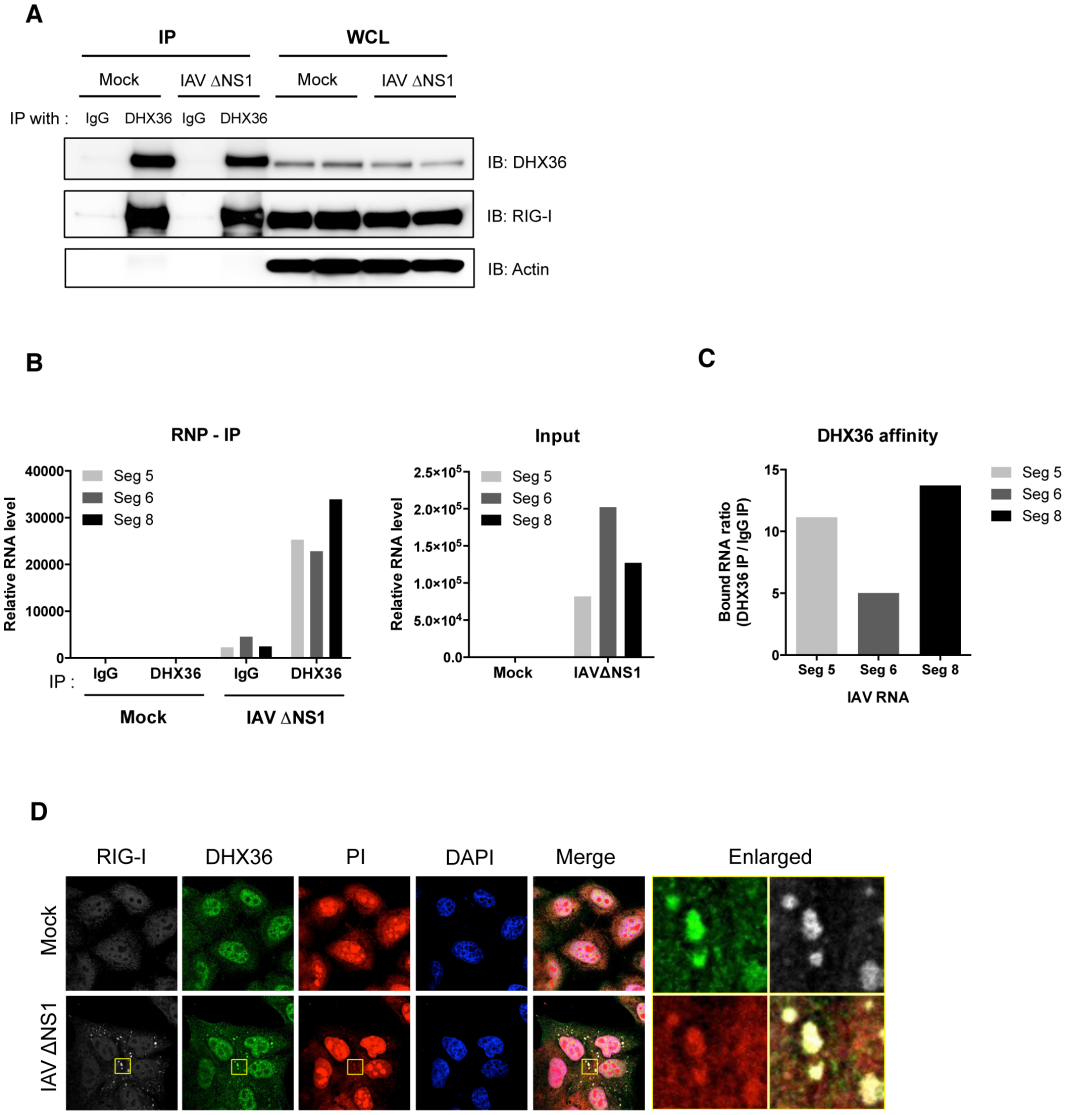
DHX36 recognizes viral RNAs. (**A–C**) HeLa cells were mock-treated or infected with IAVΔNS1. After 12 h infection, cells were collected and lysed for RNA-IP analysis. After pull-down with indicated antibodies, RNA was recovered from the RNP complex. The IP efficiency was confirmed by Western blot analysis by indicated antibodies (**A**). The level of RNAs bound to RNP complex as well as input RNAs was measured by real-time qPCR with indicated probes (**B**). DHX36 affinity was evaluated by calculating the ratio of RNAs from DHX36 IP and IgG IP (**C**). (**D**) HeLa cells were mock-treated or infected with IAVΔNS1. After 12 h infection, cells were fixed and stained for the indicated proteins. Cytoplasmic dsRNAs were detected by PI staining. Nuclei were visualized by DAPI staining. The images were taken by confocal microscopy. High magnification images of the square area are shown (Enlarged).

To further analyze whether DHX36 associates with viral RNA, we performed RNP-IP analysis using anti-DHX36 monoclonal antibody to pull down endogenous DHX36-RNA complex. Direct interaction between DHX36 and RIG-I was confirmed again by this experiment as shown in Figure 3A. We then isolated RNA from RNP complex and performed real-time qPCR to evaluate binding of DHX36 and IAV viral RNAs. We found that DHX36 associates with viral RNAs (Figure 3B). Among the viral RNAs examined, DHX36 showed the highest binding affinity to IAV segment 8 (Figure 3C). Next, we checked the localization of IAV RNA. Since IAV produces viral RNAs containing partial double-stranded panhandle-structure [1], we used a propidium iodide (PI), which specifically binds to both dsDNA and dsRNA, for staining of cytoplasmic dsRNA. IAVΔNS1 infected cells showed the cytoplasmic granules stained by PI and these foci coincided with RIG-I and DHX36 (Figure 3D). Therefore, these results suggest that DHX36 directly recognizes viral RNAs together with antiviral proteins in the avSG.

### Localization of TRIM25 to avSG

**Figure 4:**
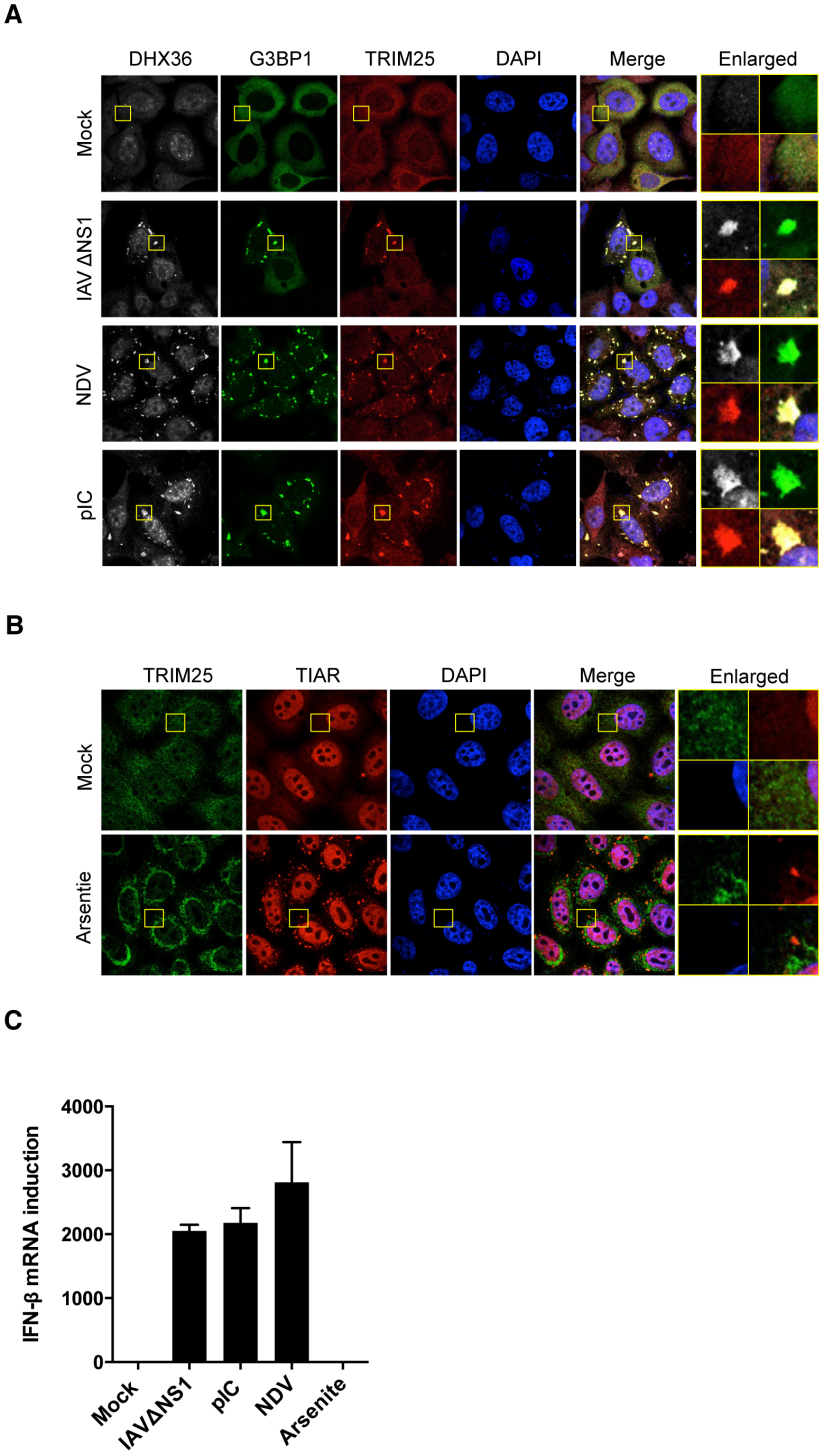
TRIM25 co-localizes with SG by virus infection or pIC transfection. (**A**) HeLa/G-G3BP cells were mock-treated or infected with viruses or transfected with pIC as indicated. After 12 h incubation, cells were fixed and stained for DHX36 and TRIM25. G-G3BP was detected by GFP fluorescence. Nuclei were visualized by DAPI staining. The images were taken by confocal microscopy. High magnification images of the square area are shown (Enlarged). (**B**) HeLa cells were mock-treated or treated with 0.5 mM of sodium arsenite for 45 min. After stimulation, cells were fixed and stained for the indicated proteins. Nuclei were visualized by DAPI staining. The images were taken by confocal microscopy. High magnification images of the square area are shown (Enlarged). (**C**) HeLa cells were mock-treated or stimulated with indicated stimuli. After incubation for 12 h for virus infection or pIC transfection, or for 45 min for sodium arsenite, cells were harvested and RNA was isolated. Level of IFN-ß gene induction was evaluated by real-time qPCR. Data are the mean ± standard error of the mean (SEM).

To further prove the functional relevance of avSG to host antiviral responses, we investigated the localization of TRIM25, which was shown to be critical for RLR signaling [27]. We used HeLa cells stably expressing GFP-tagged RasGAP SH3-domain-binding protein 1 (G3BP1), a SG marker (HeLa/G-G3BP) [11] for this purpose. TRIM25 is normally distributed diffusely in the cytoplasm. Interestingly, virus-infected or pIC-transfected cells exhibited translocation of TRIM25 to avSG together with DHX36 and G3BP1 (Figure 4A). In contrast, TRIM25 was not localized in SG induced by arsenite treatment (Figure 4B), which does not induce IFN signaling (Figure 4C), strongly suggesting that interaction between TRIM25 and antiviral proteins in the avSG is critical for the efficient antiviral signaling.

### DHX36 Is Critical for Virus-Induced avSG Formation

**Figure 5:**
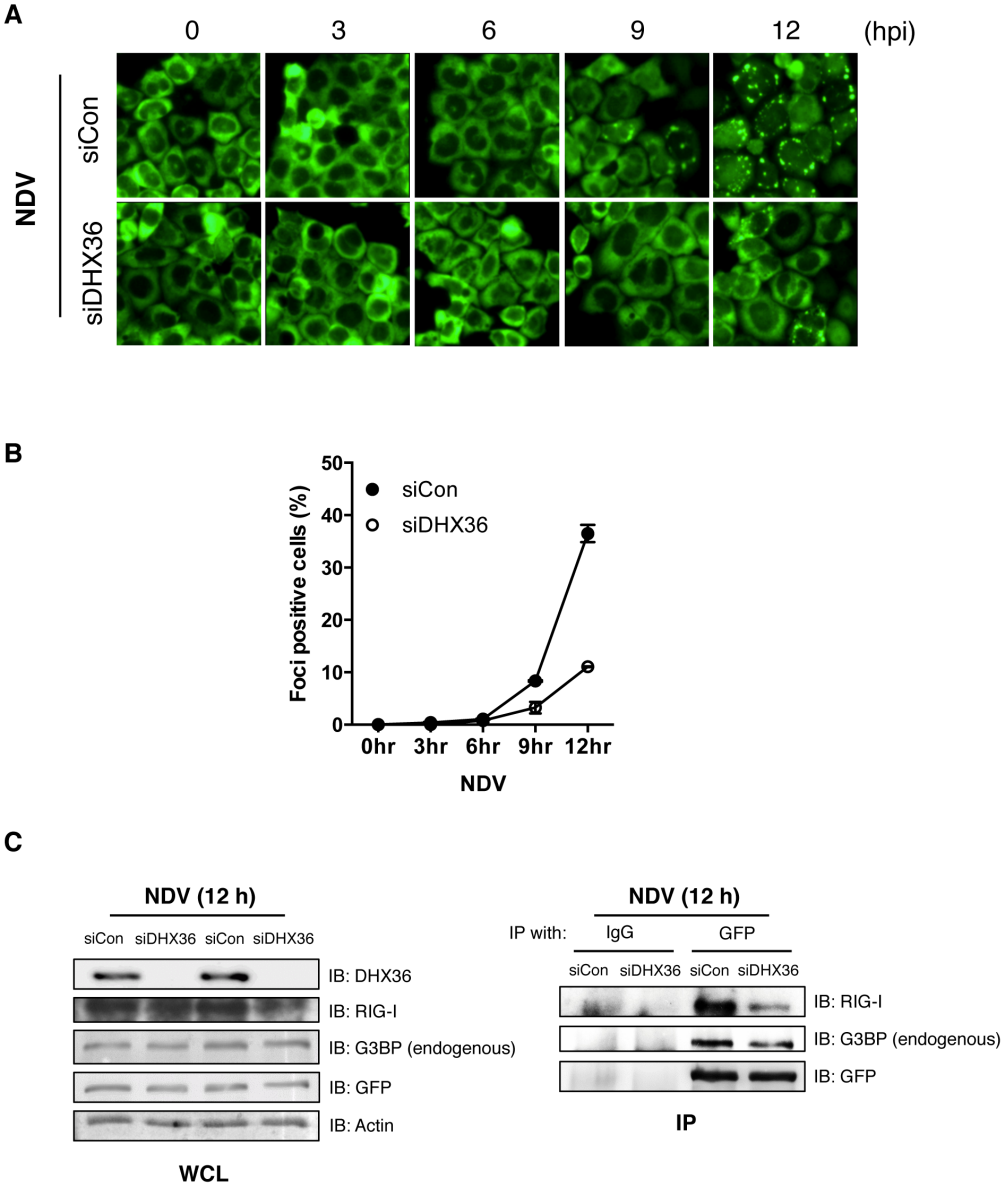
DHX36 is critical for virus-induced avSG formation. (**A–B**) HeLa/G-G3BP cells were transfected with control siRNA or siRNA targeted to DHX36 for 72 h. The cells were mock-treated or infected with NDV for indicated times. Cellular localization of GFP-G3BP was examined by florescent microscopy (**A**). Cells with GFP foci were visually analyzed and quantified (**B**). (**C**) Whole-cell extract prepared from HeLa/G-G3BP cells infected with NDV for 12 h was immunoprecipitated with either control IgG or anti-GFP antibody. Immunoprecipitates (IP) were analyzed by Western blotting with indicated antibodies. Protein expression was confirmed by immunoblotting using whole-cell lysate (WCL).

To explore the underlying mechanism of DHX36 requirement in NDV-induced IFN-ß gene activation, we knocked down DHX36 in HeLa/G-G3BP cells and examined them for avSG formation by NDV infection. NDV-induced avSG was markedly diminished by DHX36 knockdown (Figure 5A and Figure 5B); however, DHX36 deletion did not affect viral RNA production up to 12 h (Figure 1J). We previously reported that RIG-I associates with G3BP in an IAVΔNS1 infection-dependent manner [13]. Therefore, we examined the association of RIG-I and G3BP in NDV-infected cells and confirmed that these proteins physically associated in NDV-infected cells. Interestingly, this interaction was significantly attenuated by DHX36 knockdown (Figure 5C). Consistently, we also observed that IAVΔNS1-induced avSGs and antiviral signaling was inhibited by DHX36 depletion (Figure S5), confirming a critical role for DHX36 in the regulation of IFN-ß gene activation through avSGs.

### DHX36 Facilitates PKR Phosphorylation Induced by Virus or dsRNA

**Figure 6:**
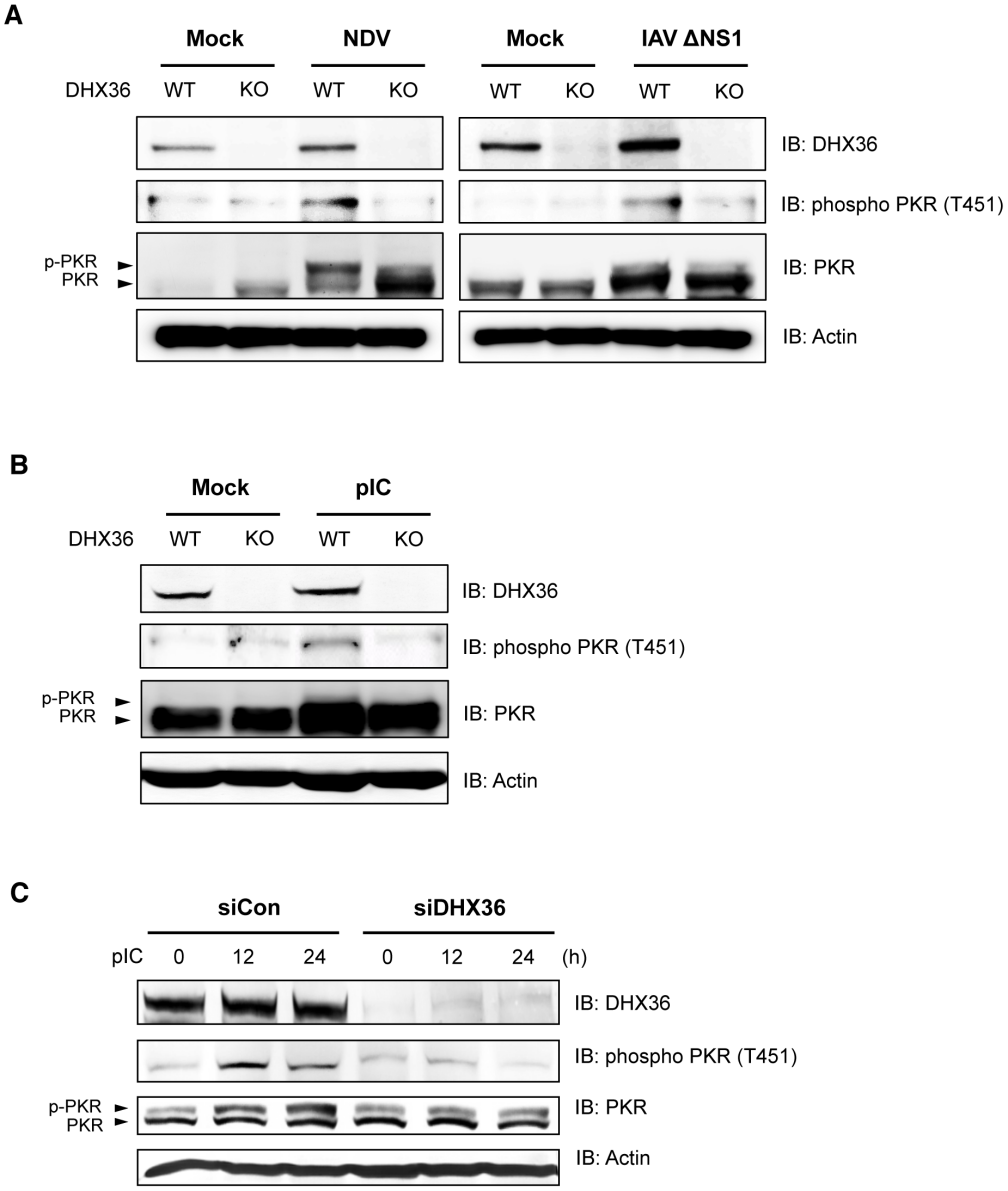
DHX36 is required for virus-induced PKR phosphorylation. (**A–B**) Tamoxifen-induced control (WT) and DHX36 knockout (KO) MEF cells were mock-treated or infected with indicated viruses (**A**) or transfected with pIC (**B**) for 12 h. Level of DHX36, phospho PKR, PKR and actin were examined by immunoblotting. (**C**) HEK293T cells were transfected with control siRNA or siRNA targeting for human DHX36. After 72 hr incubation, cells were then induced by pIC transfection for the indicated times. Whole-cell lysates were prepared and subjected to immunoblotting using indicated antibodies.

It has been hypothesized that different stresses activate one of the four distinct eIF2α kinases, including PKR, and phosphorylation of eIF2α at Ser 51 triggers the assembly of SG [28]. Upon binding of dsRNA with PKR, autophosphorylation of PKR takes place and the resultant hyperphosphorylated PKR catalyzes the phosphorylation of heterologous substrates [29], [30]. Because our data suggest that DHX36 is required for avSG formation, we further examined the effect of DHX36 deletion on PKR phosphorylation induced by viral infection. Intriguingly, virus infection induced hyperphosphorylation of PKR and this phosphorylation was markedly attenuated by DHX36 deletion (Figure 6A). pIC transfection similarly induced the phosphorylation of PKR in mouse (Figure 6B) and human cells (Figure 6C). However, depletion of DHX36 diminished the PKR phosphorylation. These results clearly suggest that DHX36 is required for efficient activation of PKR induced by dsRNA.

### Involvement of DHX36 ATPase/Helicase Activity in Autophosphorylation of PKR Induced by dsRNA

**Figure 7:**
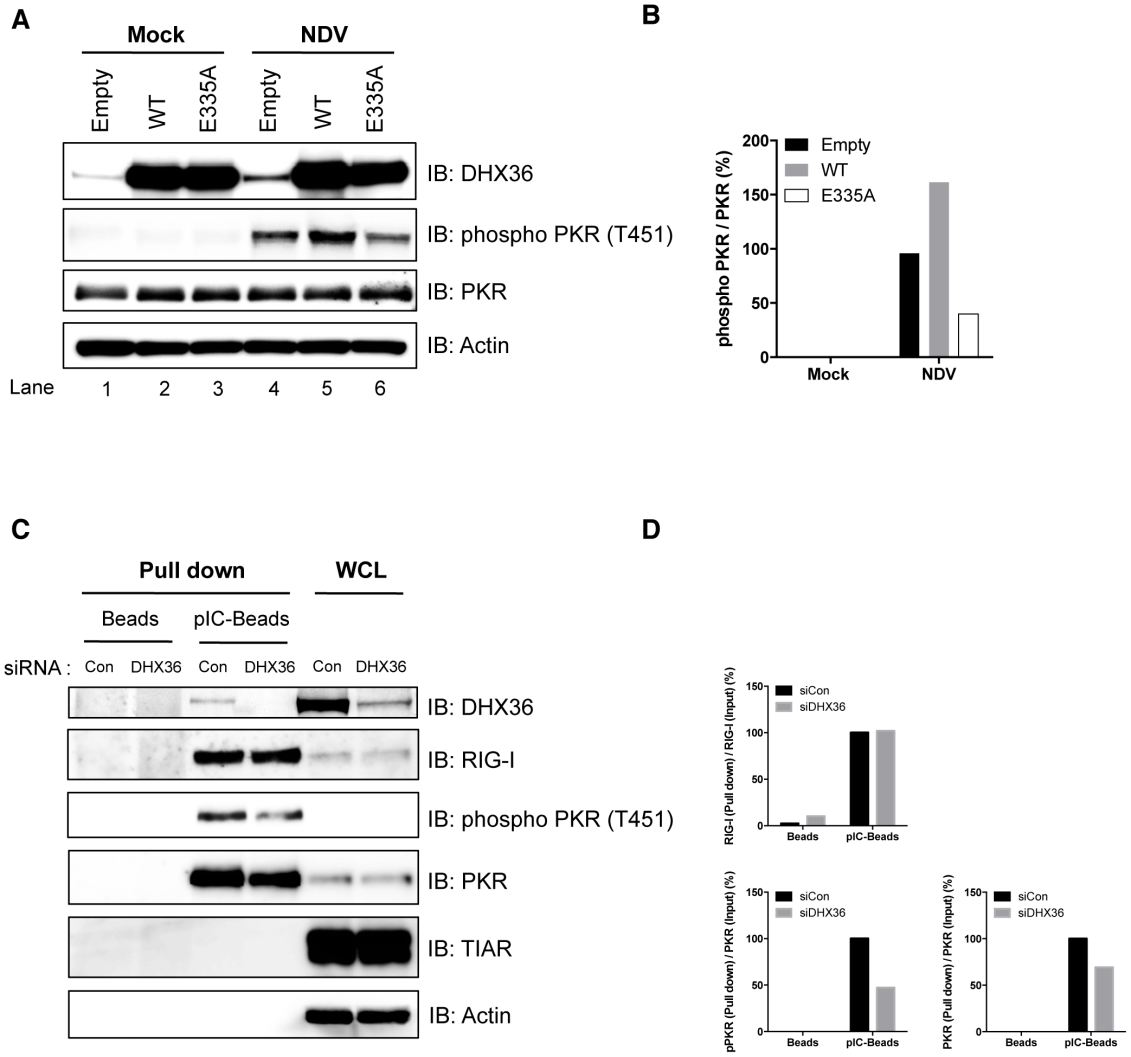
DHX36 facilitates PKR activation through its helicase/ATPase activity. (**A–B**) HEK293T cells were transfected with empty vector, expression vector for HA-DHX36, or HA-DHX36 E335A. The cells were mock-treated or infected with NDV. After 12 h infection, cells were harvested and analyzed for phosphorylation of PKR by immunoblotting (**A**). Quantification of the signals for phospho-PKR is shown (**B**). (**C–D**) HeLa cells were transfected with control siRNA or that targeting human DHX36. Whole-cell lysates were prepared and pulled down with empty- (Beads) or poly I∶C-Beads (pIC-Beads). The precipitated proteins (Pull down) were analyzed by immunoblotting using the indicated antibodies. Protein expression was confirmed by immunoblotting using whole-cell lysate (WCL) (**C**). Quantification of the signals for RIG-I, PKR or phospho-PKR is shown (**D**).

To examine the role of DHX36 in the activation of PKR, we transiently overexpressed HA-tagged DHX36 in HEK293T cells and analyzed the phosphorylation of PKR in control and NDV-infected cells. While overexpression of DHX36 alone did not induce PKR phosphorylation, NDV-induced PKR phosphorylation was augmented by ectopic expression of DHX36 (Figure 7A, lane 2 and 5 and Figure 7B). Because DHX36 is a putative RNA ATPase/helicase, we next asked whether these catalytic activities are involved in PKR activation by overexpression of an ATPase-defective DHX36 mutant, E335A [18]. Interestingly, ectopic expression of DHX36 E335A inhibited NDV-induced PKR phosphorylation, suggesting that the ATPase/Helicase activity is involved in PKR activation (Figure 7A, lane 4–6 and Figure 7B). We further asked if the physical interaction between PKR and dsRNA is affected by the presence of DHX36. To evaluate this, we pulled down RNA-protein complex from normal and DHX36-knocked down cell lysates by pIC-agarose (Figure 7C). Although the cells were not infected, PKR is activated during incubation with pIC-agarose, presumably by ATP present in the extract. In the absence of DHX36, the association of PKR, but not RIG-I, with pIC was moderately reduced with a concomitant reduction of phospho-PKR (Figure 7C and Figure 7D). Since total PKR amount was not affected by the absence of DHX36, these data suggest that DHX36 is capable of facilitating PKR association with dsRNA, hence its activation.

### DHX36 Directly Facilitates PKR Phosphorylation by dsRNA

**Figure 8:**
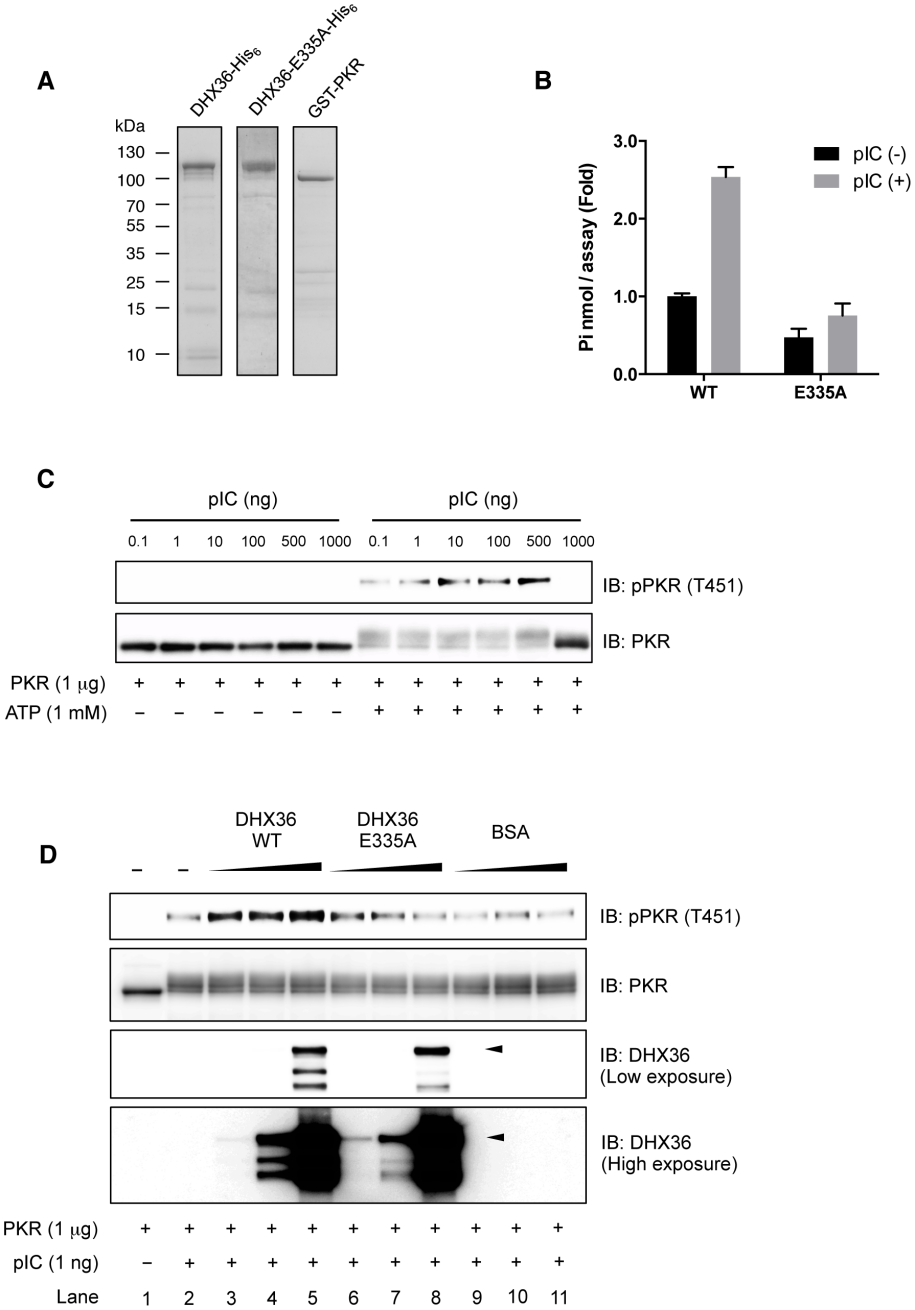
DHX36 augments PKR activation through its ATPase activity *in vitro*. Purity of recombinant proteins as examined by SDS-PAGE (**A**). Functional analysis of recombinant DHX36 WT and E335A was performed by ATPase assay using pIC (250 ng/reaction) (**B**). Autophosphorylation of recombinant PKR induced by pIC (**C**). 1 µg of recombinant PKR was mixed with buffer (–) or increasing amount of indicated proteins (1, 10 or 100 ng). Then, the mixtures were further treated with 1 ng of pIC and incubated for 5 min. After incubation, ATP was added to the mixtures and incubated for another 15 min. The level of phospho-PKR or input proteins was examined by Western blot analysis using indicated antibodies. Intact size of DHX36 WT and E335A is indicated by arrowhead (**D**).

Although our data clearly showed that DHX36 augments PKR phosphorylation in a RNA ligand-dependent manner, it is still possible that other cellular factors may also participate and contribute to PKR activation. In fact, DHX36 forms a complex with other RNA helicases for innate immune responses induced by dsRNA [22]. To clarify this issue, we further asked whether DHX36 directly facilitates PKR phosphorylation or requires other cofactors for PKR activation. To examine this, we performed an *in vitro* PKR phosphorylation assay using purified recombinant PKR, DHX36 WT and DHX36 E335A (Figure 8A). We confirmed that purified recombinant DHX36 WT, but not E335A, retained the ATPase activity (Figure 8B). We also confirmed that the recombinant PKR was functional (Figure 8C). Based on the data from Figure 8C, we chose 1 ng pIC for further analysis. As shown in Figure 8D, PKR phosphorylation was detected by pIC treatment (Figure 8D, lane 1 and lane 2). Interestingly, this PKR phosphorylation was markedly increased in the presence of DHX36 in a dose-dependent manner (Figure 8D, lane 3–5). In contrast, DHX36 E335A lost its function on facilitating the PKR phosphorylation (Figure 8D, lane 6–8) confirming the involvement of ATPase activity *in vitro*. Additionally, BSA was also tested as a non-related control for this analysis and the result did not show any significant effect (Figure 8D, lane 9–11, Figure S6). Taken together, these data indicate that DHX36 solely functions for efficient PKR activation in the presence of dsRNA through its ATPase activity.

### DHX36 Contributes to Maximal Antiviral Effect against NDV

**Figure 9:**
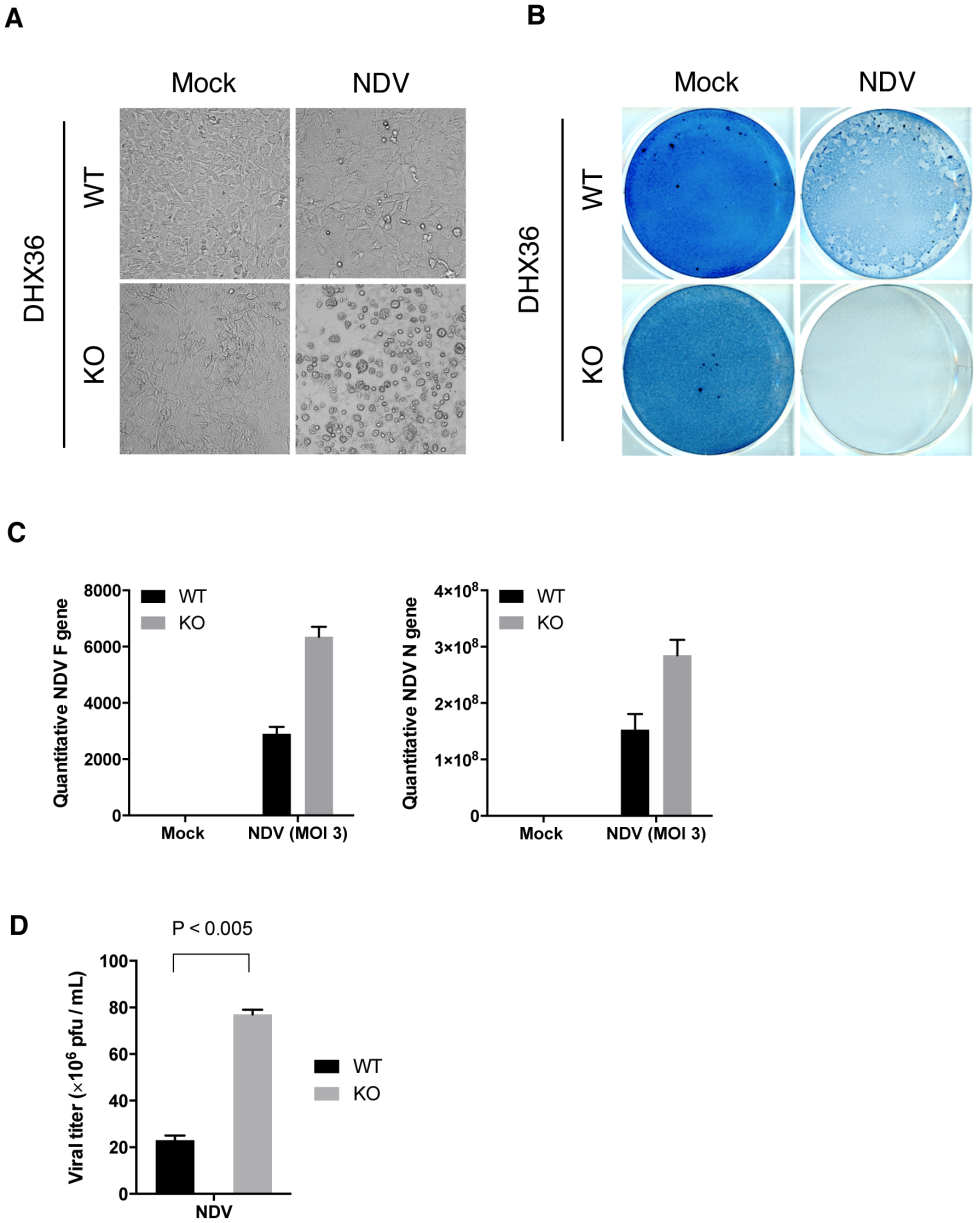
DHX36 deficiency results in enhanced cytopathic effect and propagation of NDV. (**A–D**) Tamoxifen-induced control (WT) and DHX36 knockout (KO) cells were infected with NDV for 24 h. Microscopic morphology of the cells (**A**) and amido black stained culture dish (**B**) are shown. Yield of viral RNAs after 18 h infection was examined by real-time qPCR using specific primer set for viral F and N genes (**C**). Yield of infectious viral particles were measured by plaque assay using culture supernatant from NDV infected (24 h) DHX36 WT or KO-induced MEF (**D**). Data are the mean ± standard error of the mean (SEM), P value by Student's t test is indicated.

Type I IFN plays a central role in defense against virus infection by suppressing viral yield and promoting cell survival. First, we examined the impact of deleting DHX36 on the viral cytopathic effect. WT or DHX36 KO MEFs were infected with NDV. At early time points (up to 12 h), viral RNA replication was not impaired by DHX36 knockdown (Figure 1J). However, at 24 h post infection, augmented cell death was observed in DHX36 KO cells compared to WT cells (Figure 9A and Figure 9B). Consistent with the enhanced cytopathic effect, DHX36 KO cells produced enhanced viral RNA (Figure 9C) and viral titer (Figure 9D), suggesting that DHX36 exerts its antiviral effect through the regulation of IFN production.

## Discussion

The cytoplasmic viral RNA sensor, RLR, plays a major role in detecting viral infection and triggering antiviral responses. In this report, we describe the involvement of DHX36 in sensing viral infection and subsequent activation of RIG-I. Kim et al. reported that DHX36 functions as DNA sensor in plasmacytoid dendritic cells (pDC) [21]. The reported function of DHX36 is apparently distinct from our discovery. First, we used fibroblast and epithelial cell lines, quite distinct cell types from pDC. Second, the stimuli used did not contain or produce DNA. There is another report describing DHX36 as a dsRNA sensor in myeloid dendritic cells (mDC) [22]. DHX36 appears to function upstream of TIR domain-containing adaptor inducing IFN-ß (TRIF also termed TICAM1) to activate NF-κB and IRF-3/7. Again, their observation is distinct from ours because in our system, RIG-I was essentially involved in the signal induced by dsRNA, IAVΔNS1, and NDV in the fibroblast cells [31]. It remains to be shown whether dsRNA-stimulated mDC promotes PKR activation and SG formation through DHX36.

We observed that DHX36 augments signaling by RIG-I in a stimulus-dependent manner: signaling induced by infection with IAVΔNS1, NDV and transfection of pIC is enhanced by DHX36 (Figure 1). These stimuli commonly induce cells to form SG. We and others have shown that SG formation is required to facilitate antiviral responses of host cells [11], [12], [13], [14]. Interestingly, the majority of viruses reported, including Sindbis, encephalomyocarditis, polio, adeno-, and measles viruses induce SG upon infection [11], [12], [13], [32].

**Figure 10:**
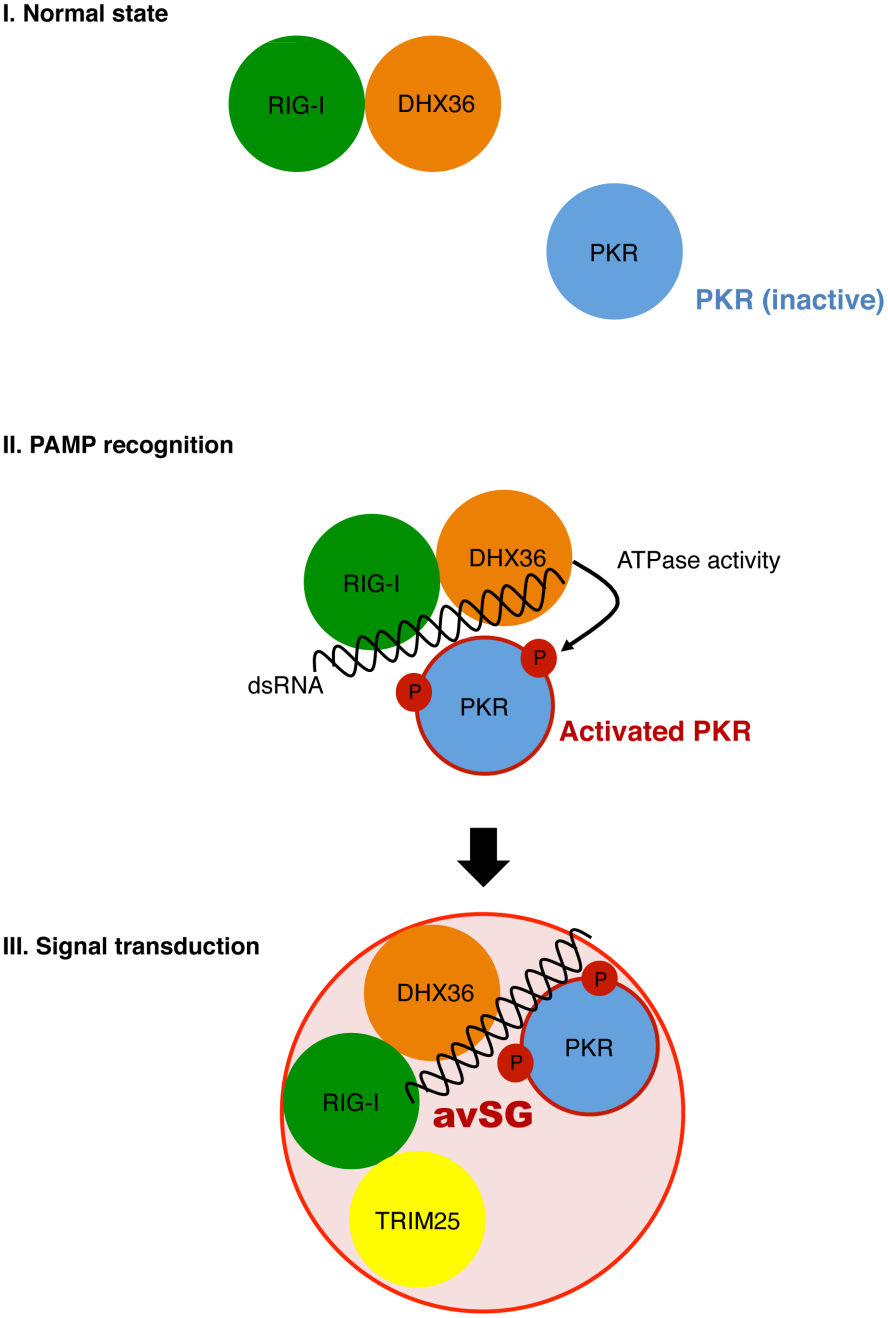
Model for functional role of DHX36 in antiviral signaling. DHX36 and RIG-I directly interact regardless of viral infection (**Normal state**). Upon viral infection, dsRNA facilitates formation of dsRNA/RIG-I/DHX36/PKR complex. DHX36 moderately increase the binding of PKR to dsRNAs as well as promoting activation of PKR through its ATPase activity (**PAMP recognition**). Finally, the activated PKR immediately induces avSG and provides a critical platform for host antiviral responses by recruitment of antiviral signal molecule, TRIM25 (**Signal transduction**).

We discovered that DHX36 facilitates the activation of PKR in the presence of dsRNA, resulting in subsequent SG formation (Figure 5–8 and also see a hypothetical model in Figure 10). DHX36 was found to physically interact with RIG-I regardless of virus infection. Under the condition of viral infection, DHX36:RIG-I complex recognizes dsRNA and interacts with PKR. Although small increase of binding of PKR to dsRNA was observed by DHX36, this does not explain the marked activation of PKR by DHX36. Interestingly, ATPase-deficient DHX36 inhibited PKR activation, suggesting that ATP hydrolysis and/or dsRNA unwinding are mechanistically involved in this regulation. In fact, DHX36 is capable of resolving several types of nucleotides containing unusual structures, such as G4-DNA and G4-RNA, and promotes further biological events [33], [34]. It is tempting to speculate that DHX36 changes conformation upon binding with dsRNA in the presence of ATP in a similar manner as another DExD/H box RNA helicase, RIG-I [35], to accelerate PKR activation with dsRNA (Figure 2D and Figure 7C). Finally, PKR activation subsequently induces avSG assembly and provides a platform of antiviral signal transduction by recruitment of TRIM25, a critical signaling molecule.

Generally, replication of RNA viruses does not take place in soluble compartments. Viruses hijack host cell structures or create a *de novo* compartment to replicate, resulting in evasion from host sensing of foreign nucleic acids. Induction of avSGs may be one of the means to force viral nucleic acids to be exposed to host immune sensors and facilitate antiviral responses. This idea is consistent with the observations that avSGs facilitate immune sensing but is not an absolute requirement.

In contrast to the viral infections examined, DHX36 did not affect IFN-ß induction by 5′ppp-cbRNA (Figure 1), even though this stimulus activates RIG-I [3]. This is partly because 5′ppp-cbRNA inadequately activates PKR and subsequent avSGs (data not shown). It remains to be explored if conferring PKR activation function to 5′ppp-cbRNA results in enhanced signaling. Alternatively, 5′ppp-cbRNA may bypass the requirement of avSG formation through an unknown mechanism. In this regard, it is tempting to hypothesize the existence of an adaptor molecule(s) that facilitates the activation of RIG-I with short 5′ppp-dsRNA to signal without avSGs. This may be relevant to the previous observation that the 5′ppp moiety is essential for RIG-I to detect short dsRNA (<50 bp) but is dispensable for long dsRNA sensing (100–500 bp) [36], [37].

In this report, we describe a novel function of DHX36 in virus-induced SG formation through PKR activation. We have been proposing a model in which SG functions as a platform to facilitate viral dsRNA recognition by RLR as well as to execute antiviral reactions [13]. The results described here further support our model and emphasize the critical involvement of the virus-induced stress response in antiviral innate immune responses. IAV and picornaviruses attenuate antiviral signaling by the activity of viral non-structural protein NS1 and 3C protease, respectively [11], [13].

## Materials and Methods

### Cell Culture and Transfection

Our current study was mainly analyzed by using DHX36 WT and KO MEF to confirm its phenotype of IFN signaling and antiviral activity. Additionally, HEK293 cells were used for the experiments of transient over-expression (Figure 2D and Figure 7A) and measuring the IFN signaling by knocking down of DHX36 (Figure 1J, Figure 6C and Figure S5 D) to support our data from KO system. HeLa cells are useful for analysis of cellular imaging and we have an excellent cellular imaging system with GFP-fused G3BP1 stably expressing HeLa cells. Thus, HeLa and GFP-G3BP1 HeLa cells were mainly utilized for monitoring of localization of antiviral proteins and avSG (Figure 2A–C, Figure 3, Figure 4 and Figure 5).

HeLa and HEK293T cells were maintained in Dulbecco's modified Eagle's medium (DMEM) supplemented with 10% fetal bovine serum (FBS) and penicillin-streptomycin (100 U/ml and 100 µg/ml, respectively). DHX36 WT or KO-inducible MEFs (GFP or CRE-GFP), and DHX36 knockdown-inducible HeLa cells were kindly provided by Dr. Nagamine (Friedrich Miescher Institute for Biomedical Research, Basel, Switzerland) [24] [38]. Briefly, DHX36 KO was induced by treatment with 1 µM tamoxifen (Sigma-Aldrich, St Louis, MO) in the culture medium for 72 h and the DHX36 KO was confirmed by Western blot analysis. For shRNA-derived DHX36 knockdown, HeLa cells were treated with doxycycline (SIGMA) at a final concentration of 1 µg/ml for 72 h. HeLa cell stably expressing EGFP-G3BP1 (HeLa/G-G3BP) was previously reported by our group [11]. The pEF-Bos-HA-DHX36 was constructed by inserting the PCR-amplified full-length DHX36 coding sequences from pEGFP-DHX36 provided by Dr. Nagamine [23] between *Bgl*II and *BamH*I sites of pEF-Bos vector. The pEF-Bos-HA-DHX36 E335A was constructed with a KOD-Plus-Mutagenesis kit (Toyobo, Osaka, Japan) using the pEF-Bos-HA-DHX36 as a template. pBos-FLAG-RIG-I WT (RIG-I 1-925) and pBos-FLAG-RIG-IΔCTD (RIG-I 1-801) were described previously [39]. All constructs were transfected into HEK293T cells with Lipofectamine 2000 (Invitrogen, Carlsbad, CA) for the experiments.

### RNA Transfection and Virus Infection

Poly I∶C was purchased from Amersham Biosciences (Arlington Heights, IL). 5′ppp-RNA (5′- pppGGAAACUGAAAGGGAGAAGUGAAAGUG -3′) [35] was synthesized by *in vitro* transcription using the AmpliScribe T7-Flash Transcription kit (Epicentre, Madison, WI). RNAs were delivered into the cells with Lipofectamine 2000 according to the manufacturer's instructions. Influenza A virus ΔNS1 strain (A/PR/8/34, ΔNS1) was originally produced by Dr. A. Garcia-Sastre (Mount Sinai School of Medicine, USA), and provided by Dr. S. Akira (Osaka University, Japan). Newcastle disease virus (Miyadera strain) was provided by Dr. Taniguchi (University of Tokyo, Japan). For virus infection, cells were washed with PBS and treated with the culture medium (‘mock-treated’) or infected with IAVΔNS1 or NDV in serum-free and antibiotic-free medium. After adsorption at 37°C for 1 h, the medium was changed and infection was continued for various periods in the presence of serum-containing DMEM.

### Enzyme-Linked Immunosorbent Assay (ELISA)

Culture supernatants were collected and subjected to ELISA with mouse IFN-ß kit (PBL Interferon Source, Piscataway, NJ) according to the manufacturer's instructions.

### Poly I∶C Pull Down, Immunoprecipitation, and Antibodies

Poly I∶C pull down assay was performed as described previously [40]. Stable transformants of FLAG-RIG-I in *rig-i* null MEFs (RIG-I null/FLAG-RIG-I) were established by transfection of a linearized plasmid (pBos-FLAG-RIG-I WT) [39], and selected with Puromycin. Immunoprecipitation was performed using whole-cell extracts from HeLa, HEK293T, HeLa/G-G3BP, or RIG-I null/FLAG-RIG-I cells (200 µg), together with 1 µg anti-FLAG (Sigma-Aldrich, St. Luis, MO, F3165), or anti-GFP (Wako, Osaka, Japan, mFX73) antibodies. After overnight incubation at 4°C, immune complexes were precipitated with protein A-Sepharose beads (Amersham Biosciences) and analyzed by SDS-PAGE and Western blotting. Anti-human and -mouse IRF-3 polyclonal antibody, and anti-RIG-I polyclonal antibody were previously described [13], [41]. Anti-human DHX36 monoclonal antibody was kindly provided by Dr. Nagamine [38]. The monoclonal antibody against Influenza NP (mAb61A5) was generated by Dr. Y. Kikuch (Iwaki Meisei University, Japan), and provided by Dr. F. Momose (Kitasato University, Japan) [42]. The anti-NDV NP monoclonal antibody was produced by Dr. Y. Nagai, and provided by Dr. T. Sakaguchi (Hiroshima University, Japan). Other antibodies used in this study were as follows: anti-phospho PKR T451 (Abcam, ab4818), anti-G3BP (Santa Cruz, sc-70283), anti-TIAR (Santa Cruz, sc-1749), anti-Actin (Sigma-Aldrich, A-1978), and anti-TRIM25 (Santa Cruz, sc-22832) antibodies. For immunofluorescence analysis, Alexa 488-, 594-, and 633- conjugated anti-mouse, anti-rabbit, or anti-goat IgG antibodies purchased from Invitrogen were used as secondary antibodies. Propidium Iodide (PI) (1∶2,000 in PBST) (Miltenyi Biotec) was used for cytoplasmic dsRNA staining.

### Poly I∶C Binding Assay

RNA binding assay was previously reported [35]. Briefly, recombinant DHX36 (1.5 µg) was mixed with 1 µg of pIC in total 10 µl of DHX36 buffer [50 mM Tris pH 7.5, 50 mM KCl, 5 mM DTT, 20% (v/v) glycerol] and incubated at 25°C for 15 min. Then, the mixture was applied to 1% agarose gel and stained with ethidium bromide (EtBr).

### RNA-Immunoprecipitation (RIP) Assay

RIP assay was performed using cell extracts from mock- or IAVΔNS1- infected HeLa cells with anti-human DHX36 monoclonal antibody by RiboCluster Profiler RIP-Assay Kit (MBL, Japan, RN1001) according to the manufacturer's recommendations. Briefly, RNA-protein complex was pulled-down with 5 µg of mouse normal IgG (Santa Cruz, sc-2025) or anti-DHX36 monoclonal antibody. Then, bound RNAs were recovered from the RNA-protein complex and used for cDNA synthesis. Real-time qPCR was further performed to evaluate the RNA level bound to DHX36 with the specific primer sets targeting IAV gene. As an internal control, human glyceraldehyde 3-phosphate dehydrogenase (GAPDH) gene was targeted. Sequence information of primers is as follows: IAV segment 5, 5′-GATGGAGACTGATGGAGAACGCCAG-3′ (Sense), 5′-AGCTGTTTTGGATCAACCGTCCCTC-3′ (antisense); IAV segment 6, 5′-GGACAGAGACTGATAGTAAG-3′ (sense), 5′-GTTAGCTCAGGATGTTGAAC-3′ (antisense); IAV segment 8, 5′-GATAACACAGTTCGAGTCTC-3′ (sense), 5′- TTTCTCGTTTCTGTTTTGGA-3′ (antisense); human GAPDH, 5′-ACTGCCAACGTGTCAGTGGT-3′ (sense), 5′-TTACTCCTTGGAGGCCATGT-3′ (antisense).

### Quantitative Reverse-Transcription PCR

Quantitative reverse transcription-PCR for IFN-ß was performed as described previously [13]. For the evaluation of viral RNA, quantitative reverse-transcription PCR was performed using SYBR green reagent (Applied Biosystems, CA, USA, 4385612) with the specific primer sets targeting NDV (F and N) or IAV (segment 5, 6 and 8) genes. As an internal control, human and mouse GAPDH gene was targeted and amplified. Sequence information of primers is as follows: NDV F, 5′-GCAGCTGCAGGGATTGTGGT-3′ (sense), 5′-TCTTTGAGCAGGAGGATGTTG-3′ (antisense); NDV N, 5′-CTCAAGAGAGGCCGCAATAC-3′ (sense), 5′-AGTGCAAGGGCTGATGTCTT-3′ (antisense); mouse GAPDH, 5′-ATCTTCTTGTGCAGTGCCAGCCTCGTCCCG-3′ (sense), 5′-AGTTGAGGTCAATGAAGGGGTCGTTGATGG-3′ (antisense). Sequence information of human GAPDH is described above.

### Immunofluorescence Microscopy and Fluorescence Live Imaging

Virus-infected HeLa or HeLa/G-G3BP cells were fixed with 4% paraformaldehyde (PFA) for 20 min at 4°C, permeabilized with 0.05% Triton X-100 in PBS for 5 min at room temperature (RT), blocked with 5 mg/ml BSA in PBST (0.04% Tween20 in PBS) for 30 min, and incubated at 4°C overnight with the relevant primary antibodies diluted in blocking buffer. The cells were then incubated with secondary antibodies at room temperature for 1 h. Nuclei were stained with 4.6-dimaidino-2-phenylinodole (DAPI) and analyzed with a confocal laser microscope, TCS-SP (Leica).

For fluorescence live imaging, HeLa/G-G3BP cells were stimulated by either NDV infection or RNA ligand transfection as described above. After 12 h stimulation, GFP fluorescence images were taken and analyzed with a fluorescence microscope system, AF6500 (Leica). The percentages of avSG-containing cells were calculated in more than five randomly chosen fields for each slide.

### RNA Interference

The siRNA negative control (Invitrogen, Cat. No. 1293-112) and siRNAs targeting human PKR (sense: 5′-UUUACUUCACGCUCCGCCUUCUCGU-3′, antisense: 5′-ACGAGAAGGCGGAGCGUGAAGUAAA-3′) and DHX36 (sense: 5′-UUCUACUGCUUACAAAUCCAGCUCC-3′, antisense: 5′-GGAGCUGGAUUUGUAAGCAGUAGAA-3′) were purchased from Invitrogen, and the siRNAs targeting human RIG-I (sense: 5′-CGGAUUAGCGACAAAUUUAUU-3′, antisense: 5′-UAAAUUUGUCGCUAAUCCGUU-3′) and mouse DHX36 (sense: 5′-CUACAACUGGCUUAUCUAUUU-3′, antisense: 5′-AUAGAUAAGCCAGUUGUAGUU-3′) were purchased from Genolution Pharmaceutical (Seoul, Korea). For knock down of target genes, siRNAs were transfected into the cells with RNAi MAX (Invitrogen) according to the manufacturer's recommendations. At 48 h or 72 h post-transfection, cells were harvested or infected with viruses for further experiments.

### Expression and Purification of Recombinant Protein

pEGST-PKR/λPP plasmid that encodes GST-fused PKR and λ protein phosphatase was kindly gifted from Dr. Takayasu Date (Kanazawa Medical University). The vector was transformed into *E. Coli* BL21(DE3)pLysS strain. The Bacteria was first grown at 37°C in LB medium containing 100 µg/ml ampicillin. Protein expression was induced by addition of 1 mM IPTG when the absorbance at 600 nm was approximately 0.4. The cells were then grown at 25°C for 16 h. After incubation, the cells were harvested by centrifugation and resuspended in PBS supplemented with protease inhibitor cocktail (EDTA free) (nacalai tesuque) and was lysed via sonication. The supernatant was collected by centrifugation and mixed with Glutathione Sepharose 4B (GE Healthcare) for 3 hr at 4°C. The protein bound to Glutathione Sepharose 4B was washed with PBS and further washed with PBS supplemented with 500 mM NaCl to remove the *E. Coli* derived nucleic acid from GST-PKR. Then the protein was eluted by elution buffer containing 50 mM Tris pH 7.5, 50 mM KCl, 5 mM DTT, 20% (v/v) glycerol, 10 mM Glutathione and was concentrated. The purity of PKR was estimated by the Gelanalyzer program (http://www.gelanalyzer.com/) and approximate purity was 80%. No contamination of *E. Coli* derived nucleic acid was confirmed by UV spectrometer.

To obtain the purified recombinant DHX36 WT and its ATPase-dead mutant, E335A, the intact human DHX36 and E335A were amplified by PCR and inserted into a pEt22b(+) (Novagen) to produce a C-terminally hexa-histidine tagged protein. The vector was transformed into *E. Coli* BL21(DE3) strain. The Bacteria was similarly grown at 37°C in LB medium containing 100 µg/ml ampicillin. The protein expression was induced by the addition of 0.01 mM IPTG when the absorbance at 600 nm was approximately 0.4. Then the cells were grown at 16°C for 16 h. The cells were harvested by centrifugation and suspended in lysis buffer containing 50 mM Tris pH 8 500 mM NaCl, 20 mM Imidazole supplemented with protease inhibitor cocktail and were lysed via sonication and centrifuged. The supernatant was suspended with a Ni-NTA (Qiagen) affinity column, then the resin was washed with lysis buffer. The protein was eluted with a gradient of 20–500 mM Imidazole dissolved in lysis buffer. The buffer containing the protein was exchanged to 50 mM Tris pH 7.5, 50 mM KCl, 5 mM DTT, 20% (v/v) glycerol and concentrated. The purity of proteins was further confirmed and approximate purity was 80%. No contamination of *E. Coli* derived nucleic acid was confirmed by UV spectrometer.

### *In vitro* PKR Phosphorylation

*In vitro* PKR phosphorylation was determined in a total volume of 10 µl containing 50 mM Tris–HCl (pH 7.5), 2 mM MgCl_2_, 50 mM KCl, 1 µg of purified GST-PKR [43], and the indicated amounts of WT or E335A hexa-histidine-DHX36 and pIC. After incubation at 30°C for 5 min, 1 µl of 10 mM ATP was supplemented and the reaction mixtures were further incubated at 30°C for 15 min. The reaction was stopped by adding 10 µl of 2× SDS sample buffer and boiled for 5 min. Samples were subjected to SDS-PAGE and the level of phosphorylated PKR was evaluated by Western blot analysis using anti-phospho PKR specific antibody. Total amount of input protein level was also confirmed by silver staining.

### ATPase Assay

0.35 mg of recombinant DHX36 WT or E335A was mock-treated or mixed with 0.25 µg pIC in a total volume of 20 µl buffer containing 20 mM Tris (pH 8.0), 1.5 mM MgCl_2_, and 1.5 mM DTT and the mixture was incubated at room temperature for 15 min. After incubation, 5 µl of 5 mM ATP was added and the mixture was further incubated at 37°C for 30 min. Finally, 5 µl of the mixture from the incubated samples was taken and diluted with 45 µl water, and 100 µl BIOMOL Green (Enzo life sciences, Farmingdale, NY) solution was added to the mixture. ATPase activity was examined by measuring the absorbance of 630 nm of the samples using a Microplate Reader 680 (Bio Rad).

### Plaque Assay

DHX36 WT and KO MEF were infected with NDV for 24 h and culture supernatant was collected. Then, virus yield in culture supernatant was determined using Hep2 cells as previously described [40].

### Accession Numbers

1. Human DHX36: NM_001114397

2. Mouse DHX36: NM_028136

3. Human RIG-I: NM_014314

4. Human PKR: NM_001135651

5. Mouse PKR: NM_011163

6. Human TIAR: NM_001033925

7. Human G3BP: NM_005754

8. Human IRF3: NM_001197122

9. Human Actin: NM_001100

10. Mouse Actin: NM_007392 NM_183274

11. Influenza A virus Segment 5 (NP): NC_002019.1

12. Influenza A virus Segment 6 (NA): NC_002018.1

13. Influenza A virus Segment 8 (NS): NC_002020.1

14. Newcastle disease virus N: NP_071466

15. Newcastle disease virus F: NP_071469

## Supporting Information

Figure S1 **Stimulus-dependent involvement of DHX36 for IFN-ß mRNA induction.** DHX36 WT or KO-induced MEF cells were infected with NDV, or transfected with 5′ppp-cbRNA. After 9 h incubation, cells were harvested and total RNA was collected. cDNA was synthesized with oligo dT as a primer. Then, IFN-ß RNA level was evaluated by real-time qPCR. Data are the mean ± standard error of the mean (SEM), P value is indicated by Student's t test.
(TIF)

Figure S2 **DHX36 regulates IFN signaling in HeLa cells.** (**A**) The conditional shRNA-derived DHX36 knockdown system was previously reported [38]. To induce shRNA expression, cells were treated with doxycycline (1 µg/ml). After 72 h incubation, cells were infected with IAVΔNS1, or transfected with pIC for 9 h. Then, cells were harvested and total RNA was collected to evaluate IFN-ß gene induction by real-time qPCR. (**B**) HeLa cells were transfected with either control siRNA or siRNA targeting DHX36 gene and incubated for 48 h. Cells were then mock-treated or transfected with pIC. After 9 h incubation, cells were harvested and total RNA was collected to examine the induction level of IFN-ß and IL6 mRNA by real-time qPCR. Knockdown efficiency was confirmed by Western blot analysis with indicated antibodies. Data are the mean ± standard error of the mean (SEM).
(TIF)

Figure S3 **Interaction between DHX36 and RIG-I in MEF.** Whole-cell extracts from *rig-I* null or Flag-RIG-I stably expressing *rig-I* null MEFs were prepared and immunoprecipitated with anti-Flag antibody. The precipitates (IP: Flag) were analyzed for mouse DHX36 and Flag by immunoblotting. Protein expression in the whole-cell lysate (WCL) was confirmed by immunoblotting.
(TIF)

Figure S4 **DHX36 directly binds to poly I∶C.** Recombinant DHX36 (1.5 µg) was mixed with pIC (1 µg) and separated on 1% agarose gel. The gel was stained with ethidium bromide (EtBr) and visualized by ultra violet illumination.
(TIF)

Figure S5 **DHX36 positively regulates IAVΔNS1-induced avSG and antiviral signaling.** (**A–C**) HeLa cells were transfected with siCon or siDHX36. After 48 h incubation, cells were infected with IAVΔNS1 for 12 h. Then, cells were fixed and stained for IRF-3, IAV NP and TIAR (**A**). The percentage of cells showing cytoplasmic foci was determined by cell counting (**B**). The percentage of cells with nuclear IRF-3 after IAVΔNS1 infection was counted (**C**). (**D**) 293T cells were transfected with siCon or siDHX36 and incubated for 48 h. Then, cells were transfected with luciferase reporter gene under regulation by 8 tandem repeats of IRF binding sites (C1B-Luc). After 24 h transfection, cells were mock-treated or infected with IAVΔNS1 for 12 h. Reporter gene expression was determined by Dual-Luciferase Reporter Assay System (Promega, Madison, WI) according to the manufacturer's instructions. As an internal control, the Renilla Luciferase construct pRL-TK was used. Data are the mean ± standard error of the mean (SEM).
(TIF)

Figure S6 **Confirmation of the protein level by silver staining in the phosphorylation assay.** Samples used *in vitro* phosphorylation assay (Figure 8D) were subjected to SDS-PAGE and the gel was stained by standard silver staining to confirm the amount of proteins. Each protein used for *in vitro* phosphorylation was indicated by arrowhead.
(TIF)
