# Supplementary figures and images for: DHX36 Enhances RIG-I Signaling by Facilitating PKR-Mediated Antiviral Stress Granule Formation
Source: PLoS Pathog. 2014 Mar 20;10(3):e1004012. doi: 10.1371/journal.ppat.1004012 (PMC3961341; doi:10.1371/journal.ppat.1004012)

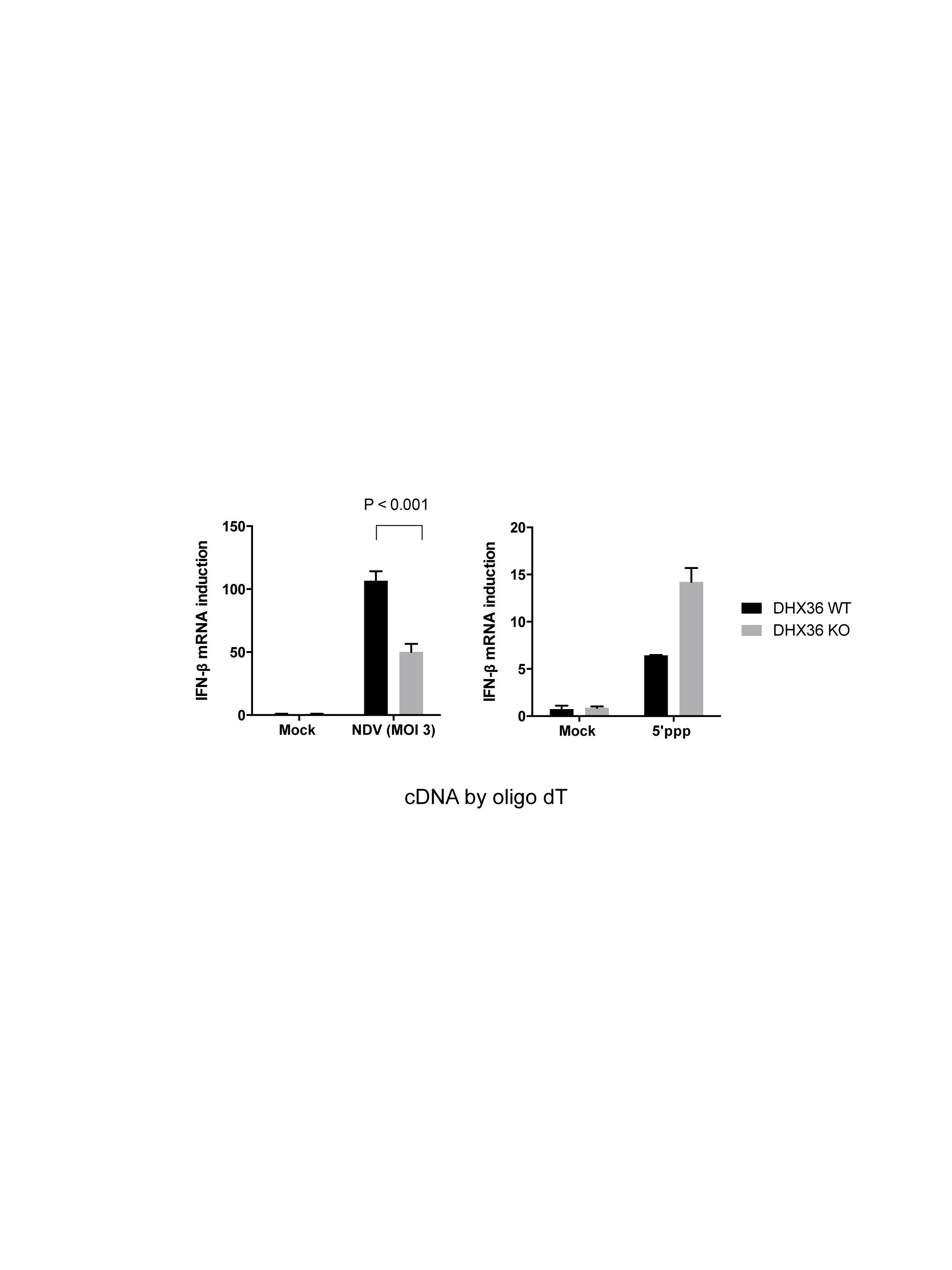

Supplement: Figure S1 — Stimulus-dependent involvement of DHX36 for IFN-ß mRNA induction. DHX36 WT or KO-induced MEF cells were infected with NDV, or transfected with 5′ppp-cbRNA. After 9 h incubation, cells were harvested and total RNA was collected. cDNA was synthesized with oligo dT as a primer. Then, IFN-ß RNA level was evaluated by real-time qPCR. Data are the mean ± standard error of the mean (SEM), P value is indicated by Student's t test. (TIF) [file ppat.1004012.s001.tif]

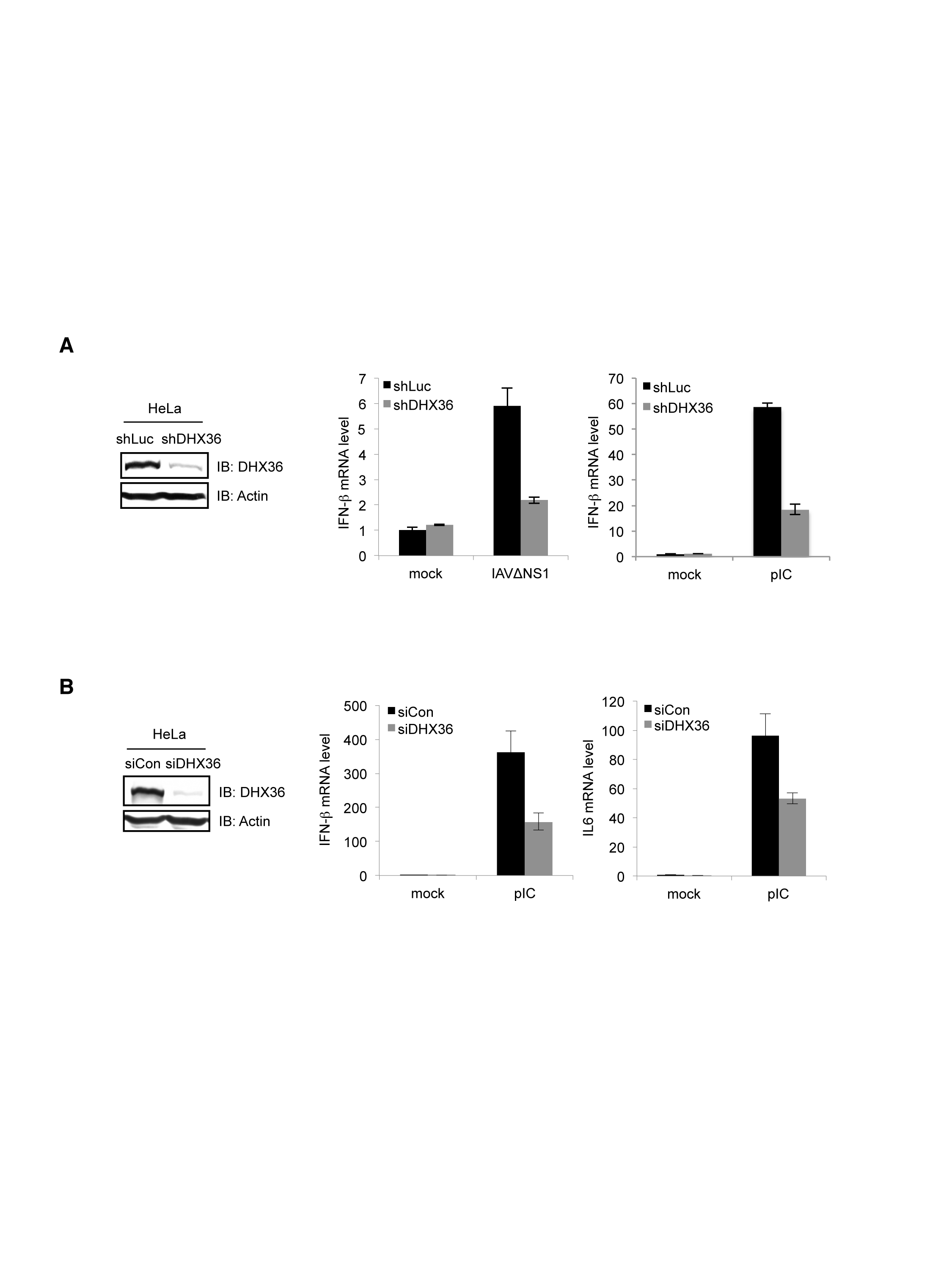

Supplement: Figure S2 — DHX36 regulates IFN signaling in HeLa cells. (A) The conditional shRNA-derived DHX36 knockdown system was previously reported [38]. To induce shRNA expression, cells were treated with doxycycline (1 µg/ml). After 72 h incubation, cells were infected with IAVΔNS1, or transfected with pIC for 9 h. Then, cells were harvested and total RNA was collected to evaluate IFN-ß gene induction by real-time qPCR. (B) HeLa cells were transfected with either control siRNA or siRNA targeting DHX36 gene and incubated for 48 h. Cells were then mock-treated or transfected with pIC. After 9 h incubation, cells were harvested and total RNA was collected to examine the induction level of IFN-ß and IL6 mRNA by real-time qPCR. Knockdown efficiency was confirmed by Western blot analysis with indicated antibodies. Data are the mean ± standard error of the mean (SEM). (TIF) [file ppat.1004012.s002.tif]

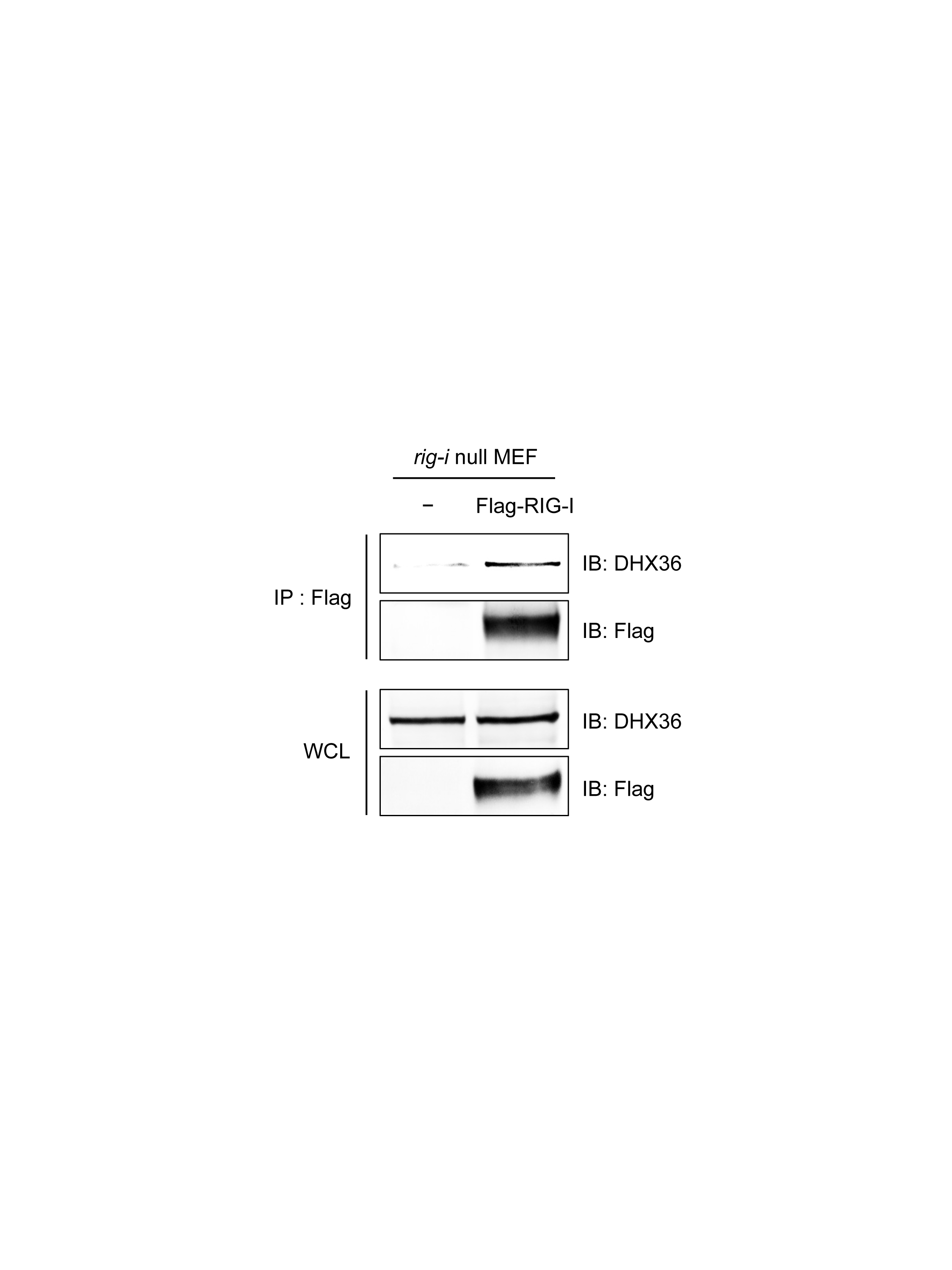

Supplement: Figure S3 — Interaction between DHX36 and RIG-I in MEF. Whole-cell extracts from rig-I null or Flag-RIG-I stably expressing rig-I null MEFs were prepared and immunoprecipitated with anti-Flag antibody. The precipitates (IP: Flag) were analyzed for mouse DHX36 and Flag by immunoblotting. Protein expression in the whole-cell lysate (WCL) was confirmed by immunoblotting. (TIF) [file ppat.1004012.s003.tif]

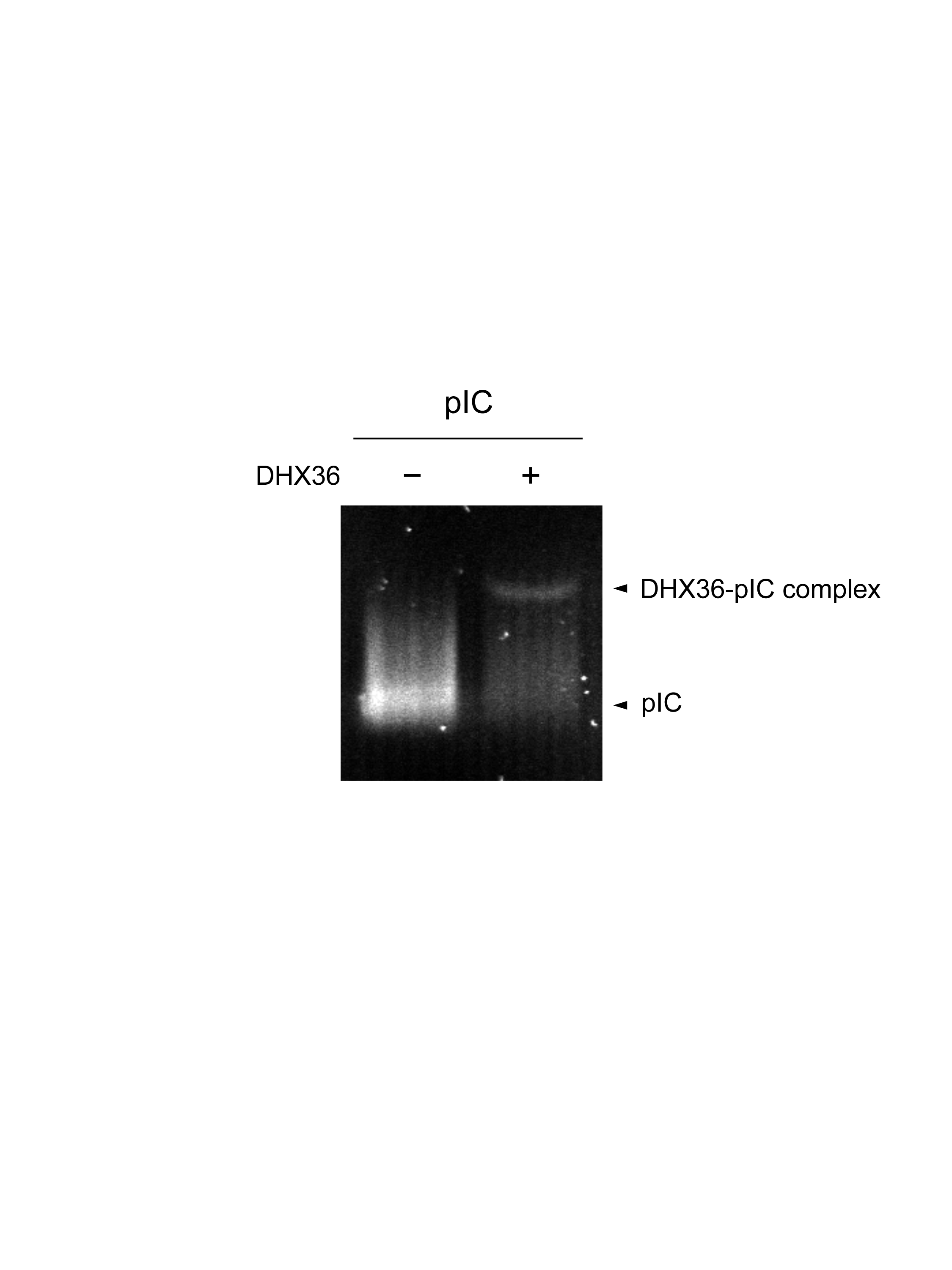

Supplement: Figure S4 — DHX36 directly binds to poly I∶C. Recombinant DHX36 (1.5 µg) was mixed with pIC (1 µg) and separated on 1% agarose gel. The gel was stained with ethidium bromide (EtBr) and visualized by ultra violet illumination. (TIF) [file ppat.1004012.s004.tif]

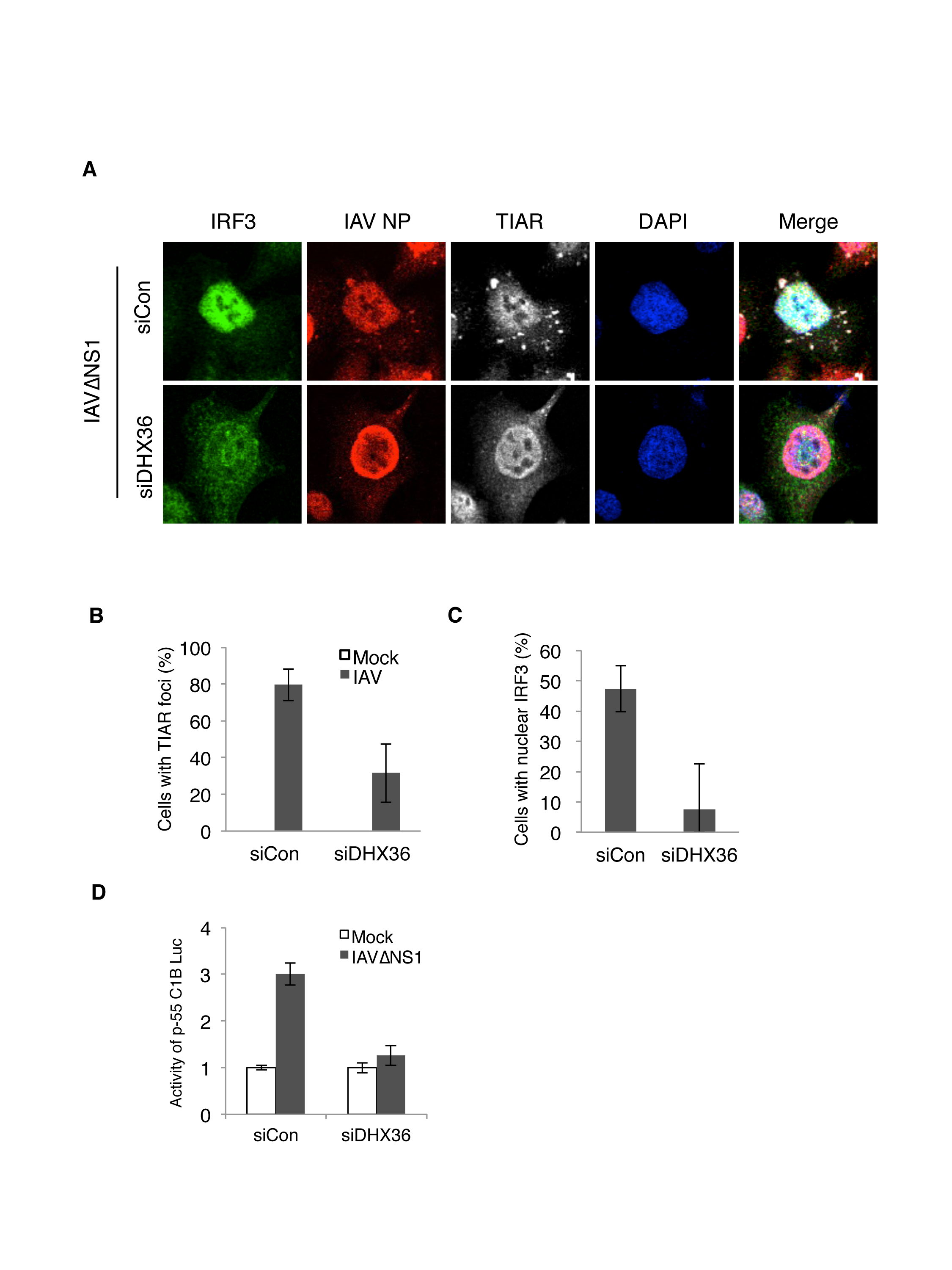

Supplement: Figure S5 — DHX36 positively regulates IAVΔNS1-induced avSG and antiviral signaling. (A–C) HeLa cells were transfected with siCon or siDHX36. After 48 h incubation, cells were infected with IAVΔNS1 for 12 h. Then, cells were fixed and stained for IRF-3, IAV NP and TIAR (A). The percentage of cells showing cytoplasmic foci was determined by cell counting (B). The percentage of cells with nuclear IRF-3 after IAVΔNS1 infection was counted (C). (D) 293T cells were transfected with siCon or siDHX36 and incubated for 48 h. Then, cells were transfected with luciferase reporter gene under regulation by 8 tandem repeats of IRF binding sites (C1B-Luc). After 24 h transfection, cells were mock-treated or infected with IAVΔNS1 for 12 h. Reporter gene expression was determined by Dual-Luciferase Reporter Assay System (Promega, Madison, WI) according to the manufacturer's instructions. As an internal control, the Renilla Luciferase construct pRL-TK was used. Data are the mean ± standard error of the mean (SEM). (TIF) [file ppat.1004012.s005.tif]

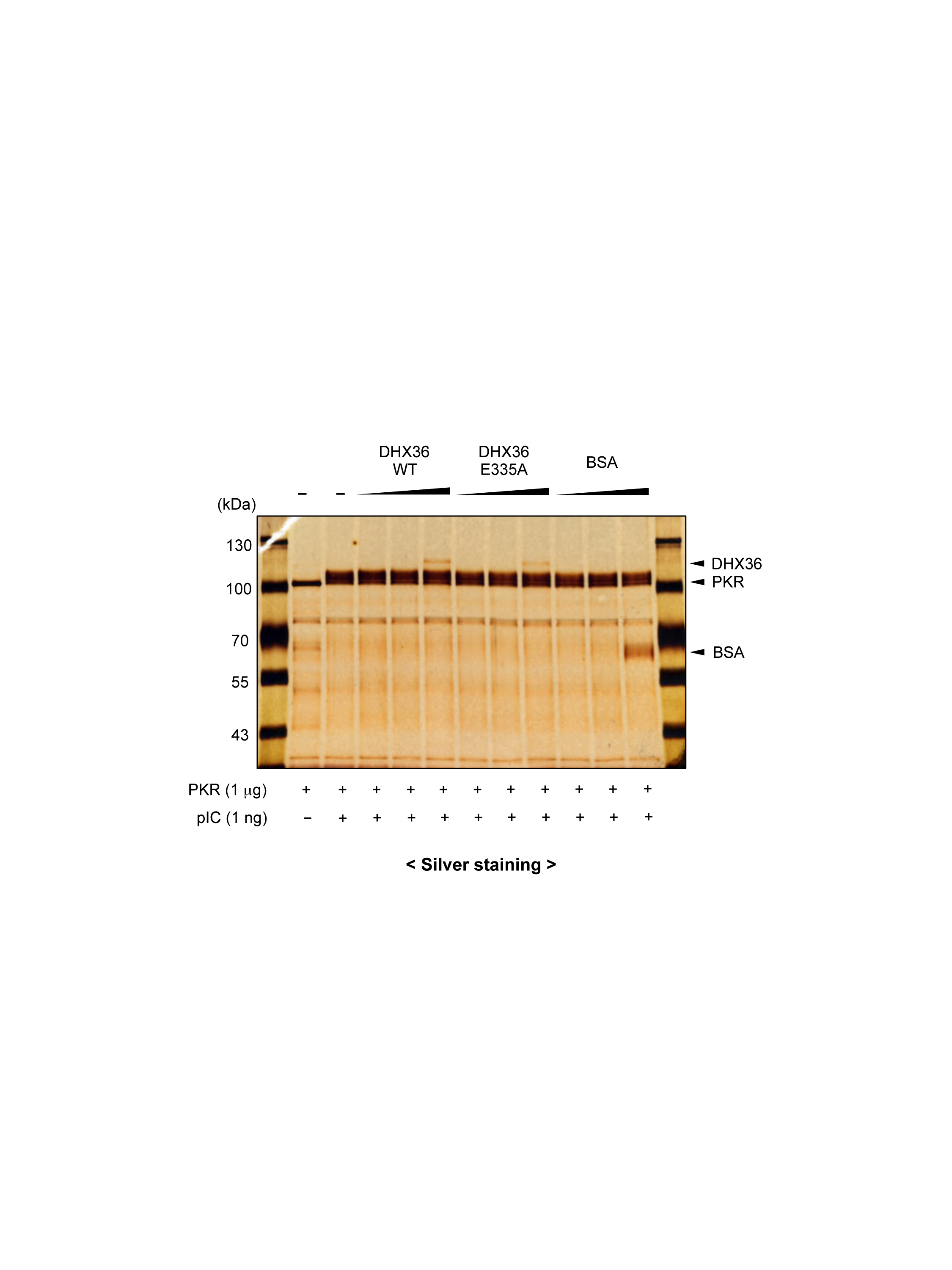

Supplement: Figure S6 — Confirmation of the protein level by silver staining in the phosphorylation assay. Samples used in vitro phosphorylation assay (Figure 8D) were subjected to SDS-PAGE and the gel was stained by standard silver staining to confirm the amount of proteins. Each protein used for in vitro phosphorylation was indicated by arrowhead. (TIF) [file ppat.1004012.s006.tif]
